# A Comparison of the Molecular Pharmacological Properties of Current Short, Long, and Ultra‐Long‐Acting β_2_‐Agonists Used for Asthma and COPD

**DOI:** 10.1002/prp2.70154

**Published:** 2025-08-31

**Authors:** Richard G. W. Proudman, Jillian G. Baker

**Affiliations:** ^1^ Cell Signalling, COMPARE, School of Life Sciences, C Floor Medical School, Queen's Medical Centre, University of Nottingham Nottingham UK; ^2^ Respiratory Medicine, Sherwood Forest Hospitals NHS Trust, King's Mill Hospital Nottinghamshire UK; ^3^ Respiratory Medicine, Nottingham University NHS Trust, Queen's Medical Centre Nottingham UK

## Abstract

β‐agonists have been used in asthma for 120 years. There are two recent changes: ultra‐long‐acting agonists for COPD and new asthma guidelines recommending formoterol/ICS inhalers phasing out short‐acting salbutamol inhalers. Few studies directly compare the molecular pharmacological properties of short (salbutamol, terbutaline, fenoterol), long (formoterol, salmeterol), and ultra‐long‐acting (indacaterol, olodaterol, vilanterol) β_2_‐agonists. Here, the in vitro molecular pharmacological properties of affinity, selectivity, intrinsic efficacy, and duration of β_2_‐agonists at human β_2_ and β_1_‐adrenoceptors and the 4 β_2_‐polymorphisms stably expressed in CHO cells were directly compared using radioligand binding and functional studies. Whilst short‐acting drugs were similar, there was huge variation and complete overlap in the molecular pharmacological properties of drugs labeled as long and ultra‐long‐acting β_2_‐agonists. Salmeterol and vilanterol were highly β_2_‐selective (> 1000‐fold) whereas indacaterol was similar to salbutamol (40‐fold). Formoterol and indacaterol were the most efficacious, whereas salmeterol had the longest duration of binding. Salmeterol and vilanterol utilize a β_2_‐specific exosite (β_2_‐H296‐K305) for high affinity and selectivity (that does not affect intrinsic efficacy or duration) whilst the β_2_‐selectivity of formoterol and olodaterol resides elsewhere. Duration of binding closely correlated with lipophilicity. β_2_‐polymorphisms had no substantial effect on β_2_‐agonist properties. Comparison with other β‐ligands suggests that affinity and duration could both be improved further. However, given the very wide range of molecular pharmacological properties of β‐agonists that are clinically effective and widely used, non‐pharmacological properties (physiochemical, patient factors, devices and combination inhaler availability) may be as important in final clinical patient outcomes as the molecular pharmacological properties of the individual β_2_‐agonists themselves.

**Abbreviations**
cAMP: cyclic adenosine 3′,5′‐monophosphate
CHO: Chinese hamster ovary
COMT: catecholamine o‐methyltransferase
COPD: chronic obstructive pulmonary disease
DPI: dry powder inhaler
EL: extracellular loop
GINA: Global Initiative for Asthma
IBMX: 3‐isobutyl‐1‐methylxanthine
ICS: inhaled corticosteroid
LABA: long‐acting β_2_‐agonist
LAMA: long‐acting muscarinic antagonist
MART: maintenance and reliever therapy
MDI: metered dose inhaler
PBS: phosphate buffered saline
SABA: short‐acting β_2_‐agonist
sfm: serum free media
SPAP: secreted placental alkaline phosphatase
TM: transmembrane
uLABA: ultra long‐acting β_2_‐agonist
WT: wildtype

## 1 Introduction

β‐agonists have been used in the management of asthma for 120 years [1]. Following experiments with adrenal extracts [2], adrenaline (isolated by Jokchi Takamine in 1901) was first used in asthma in 1903 [3, 4] where patients felt dramatic relief within minutes [5, 6]. Kahn [7] showed that adrenaline caused bronchial smooth muscle relaxation, and although adrenaline was first used as an aerosol in 1910 [8], it was the 1930s development of commercially available, although rather fragile, glass nebulizers for adrenaline to be regularly administered as an aerosol, and from 1956, in metered dose inhalers (MDI; [5, 9]).

By the 1960s, adrenaline and (the more β‐selective catecholamine) isoprenaline inhaler use was widespread but, by the late‐1960s, became linked to a spike in asthma deaths (see [1, 10, 11], and references therein). The cause of the excess deaths remains contested, but explanations include adrenaline/isoprenaline “toxicity” causing cardiac arrythmias (more pronounced in hypoxia of an asthma “attack”), receptor desensitization and tachyphylaxis, overuse/over reliance on the new inhalers, lack of urgency seeking medical help, and/or underuse of inhaled corticosteroids (ICS). A second wave of asthma deaths occurred in New Zealand, beginning in 1976, following the introduction of fenoterol inhalers (an efficacious metabolically stable derivative of adrenaline; [1, 11] and references therein).

Salbutamol [12] and terbutaline [13] are non‐catechol derivatives of adrenaline with distinct pharmacological benefits. Both are partial agonists in relation to adrenaline/isoprenaline (with less potential for receptor internalization, desensitization, and tachyphylaxis), are more β_2_‐selective, causing bronchodilation with less cardiac effects, are more stable, and are COMT‐insensitive, altogether resulting in more sustained bronchodilation with less blood pressure and heart rate effects ([12]; Baker and Shaw and references therein). Salbutamol is currently the main rescue treatment for asthma and COPD worldwide (short‐acting β_2_‐agonist, SABA) both as the “blue inhaler” used by millions daily and nebulizers used at home and in hospitals, and importantly has never been linked to an increase in deaths.

Salmeterol [14] arose from a targeted program to create long‐acting β_2_‐agonists (LABAs) to overcome the need for repeat salbutamol (SABA) dosing required to maintain day and nighttime symptoms. Formoterol was developed from a program developing β_2_‐selective agonists [15] and was initially considered short‐acting, although by serendipity it was found to have a longer duration in inhalational studies [6, 16]. Inhalers containing these LABAs have become the mainstay of asthma maintenance therapy as twice‐daily inhalers in combination with ICS. Ultra‐long‐acting β_2_‐agonists (uLABAs) have more recently been developed that require only once‐daily inhaled dosing (indacaterol, [17]; olodaterol, [18]; vilanterol, [19]) although these are currently only licensed for use in COPD and not asthma.

Following more recent clinical trials, National and International guidelines for asthma have recently changed [20, 21]. Guidelines now suggest MART (maintenance and reliever therapy), with a single LABA formoterol+ICS inhaler for as‐needed rescue therapy and regular twice daily maintenance therapy and thus replacing SABA “blue” salbutamol reliever inhalers and “brown” maintenance ICS inhalers.

There are few studies that directly compare the molecular pharmacological properties of SABAs (salbutamol, terbutaline), LABAs (formoterol, salmeterol) and particularly the uLABAs (indacaterol, olodaterol, vilanterol). This study therefore directly compared, in vitro, the molecular pharmacological properties of affinity, selectivity, intrinsic efficacy, and duration for these β_2_‐agonists at the human β_2_ and β_1_‐adrenoceptor. Given previous mixed reports with the naturally occurring β_2_‐polymorphisms on molecular pharmacological and clinical responses [22], pharmacological responses from the 4 β_2_‐polymorphic variants were also examined. Finally, as salmeterol's high selectivity is known to occur via a specific β_2_‐adrenoceptor exosite [23, 24], the interaction of other β_2_‐agonists with this exosite was also investigated.

## 2 Materials and Methods

### 2.1 Materials

Molecular biology reagents were from Promega (Madison, WI, USA) and Top 10F competent cells were from Life Technologies (Paisley, UK). The QuikChange mutagenesis kit was from Stratagene (La Jolla, CA). OPTIMEM (11058‐021) was from Gibco (Thermo‐Fisher) and Lipofectamine (18324‐020), G418 (=neomycin, 329 400 050), hygromycin (H044) and indacaterol (464652500) were from Thermo‐Fisher Scientific (Massachusetts USA). ^3^H‐CGP12177, ^3^H‐adenine, and ^14^C‐cAMP were from Amersham International (Buckinghamshire, UK) and Microscint 20 and Ultima Gold XR scintillation fluid were from PerkinElmer (Shelton, CT, USA). AG 50 W‐4X resin was from Bio‐Rad (Hertfordshire, UK). L‐glutamine (G7513), Dulbecco's modified Eagle's medium nutrient mix F12 (DMEM/F12: D6421), fetal calf serum (F7524), diethanolamine (D8885), p‐NPP (71768), CGP12177 (C125), CGP20712A (C231), fenoterol (F1016), IBMX (I5879), ICI118551 (I127), isoprenaline (I5627), propranolol (P0884) and terbutaline (T2528) were from Sigma Chemicals (Poole, Dorset, UK). Vilanterol (S3727) was from Sellakchem (Houston, USA) and olodaterol (A15761) from Adooq Bioscience. Formoterol (1440), salbutamol (0634), and salmeterol (1660) were from Tocris Life Sciences (Avonmouth, UK).

### 2.2 Molecular Biology

The wildtype β_2_‐adrenoceptor (β_2_‐WT) was initially described with arginine (R) at position 16, glutamate (E) at position 27, valine (V) at position 34, and threonine (T) at position 164. Two common N‐terminus polymorphisms (glycine (G) at position 16, glutamine (Q) at position 27) and 2 rare TM polymorphisms (TM1 methionine (M) at position 34 and TM4 isoleucine (I) at position 164) have been identified [25, 26]. β_2_‐WT in pcDNA3.1 was obtained from the Missouri S&T cDNA Resource Centre (www.cdna.org), subcloned as a HindIII/XbaI fragment into pcDNA3.1, and the sequence confirmed by DNA sequencing. Mutations were generated using QuikChange mutagenesis [27]. After subcloning in Top 10F competent cells, each mutant β_2_ cDNA was excised on Hind III/XbaI and subcloned into native pcDNA3.1 containing a neomycin selection marker. All mutations and sequences were confirmed by DNA sequencing using the School of Life Sciences Sequencing Facility. Thus, the human β_2_‐WT is R16; E27; V34; T164 or REVT, and the polymorphic variants are β_2_‐gly16 = GEVT; β_2_‐gln27 = RQVT; β_2_‐met34 = REMT; and β_2_‐ile164 = REVI.

### 2.3 Cell Culture

All CHO cells (CHO‐K1 RIDD: CVCL_0214) were grown in Dulbecco's modified Eagle's medium nutrient mix F12 (DMEM/F12) containing 10% foetal calf serum and 2 mM L‐glutamine in a 37°C humidified 5% CO_2_: 95% air atmosphere. CHO cells stably expressing a CRE‐SPAP reporter gene [27] were transfected with the β_2_‐WT, β_2_‐gly16, β_2_‐gln27, β_2_‐met34, β_2_‐ile164, or the β_2_‐H296K‐K305D construct [23] using lipofectamine and OPTIMEM and selected for 3 weeks using resistance to neomycin (1 mg/mL for the receptor) and hygromycin (200 μg/mL for the CRE‐SPAP reporter). After selection, all antibiotics were removed and cells were grown in the absence of any antibiotics for the rest of the study. Single clones were identified by dilution cloning and expanded to generate stable cell lines. Given that β_2_‐adrenoceptors are thought to be expressed at about 70fmol/mg protein in human airway smooth muscle cells [28], cell lines with a relatively low level of β_2_‐adrenoceptor were initially selected; although once bulked up for experimental use, a few differences in receptor expression in the polymorphic cell lines emerged. The CHO‐β_1_‐CRE‐SPAP cells were from [29]. Cell lines were intermittently screened throughout the study for mycoplasma and were negative.

### 2.4 ^3^H‐CGP12177 Whole Cell Binding

Cells were grown to confluence in white‐sided, tissue culture treated 96‐well view plates. ^3^H‐CGP12177 whole cell competition binding was performed as previously described [29]. The *K*_D_ for ^3^H‐CGP12177 was determined from saturation experiments using 0.003–14.38 nM ^3^H‐CGP12177. Media was removed from all wells, 100 μL serum free media (sfm = DMEM/F12 containing and 2 mM L‐glutamine) or 100 μL sfm containing 20 μM propranolol (to define non‐specific binding) added to the wells, immediately followed by 100 μL ^3^H‐CGP12177 in sfm (1:2 dilution in well, quadruplicate wells/plate). After 2 h at 37°C, plates were washed with 2 × 200 μL 4°C PBS, 100 μL Microscint 20 was added to each well, and after several hours in the dark, counted on a Topcount for 2 min per well.

To determine the affinity (*K*_D_) of other ligands, competition experiments were conducted. Media was removed from confluent cells, 100 μL competing ligand (at twice final concentration, triplicate wells/plate) added to each well immediately followed by 100 μL fixed concentration of ^3^H‐CGP12177 to give final ^3^H‐CGP12177 concentrations in the range of 0.43–3.03 nM. After 2 h at 37°C, the cells were washed and plates processed as above. Propranolol (10 μM) was used to define non‐specific binding.

A relative measure of the duration of receptor binding was achieved as described in [23]. Cells were incubated with 100 μL competing ligand and 100 μL ^3^H‐CGP12177 for 2 h as above (control plate) or 100 μL competing ligand +100 μl sfm for 2 h (wash plate = competing ligand alone). After 2 h, the control plate was washed as above while the wash plate was washed with 2 × 200 μL warm sfm. 100 μ ^3^H‐CGP12177 + 100 μL sfm was added to the wells = ^3^H‐CGP12177 alone (except non‐specific binding wells when propranolol re‐added) and incubated for 2 h. After this, the plates were washed with cold PBS and Microscint was added as above. Total and non‐specific binding were determined in 6 wells in all plates, and as the duration plate had more washes than the control plate (with more potential for cell loss), the data were normalized to the total and non‐specific binding values measured in each plate.

### 2.5 ^3^H‐cAMP Accumulation

Cells were grown to confluence in sterile, clear plastic, tissue culture treated 48‐well plates then pre‐labeled with ^3^H‐adenine by 2 h 37°C incubation with 2 μCi/mL ^3^H‐adenine in media (0.5 mL per well). Cells were washed with 1 mL sfm per well, 0.5 mL sfm containing 1 mM IBMX (3‐isobutyl‐1‐methylxanthine) added to each well, followed by agonists (in 5 μL sfm, triplicate wells/plate) and incubated for 5 h at 37°C. The assay was performed by adding 50 μL concentrated HCl per well, the plates frozen, thawed, and ^3^H‐cAMP separated from other ^3^H‐nucleotides by sequential column chromatography as in [30]. Basal response and that to a control maximum (10 μM isoprenaline) were measured in 4 wells of each per plate.

### 2.6 CRE‐SPAP Gene Transcription

CRE‐SPAP production was measured as in [31]. Confluent cells in clear‐sided, tissue culture treated 96‐well plates were serum‐starved (media removed and replaced by 100 μL sfm per well) for 24 h before experimentation. The sfm was removed and 100 μL fresh sfm (with or without a final concentration of antagonist) added per well. Agonist (in 10 μL, triplicate wells) was then added, and the plates were incubated for 5 h at 37°C. Sfm and all drugs were removed, 40 μL sfm was added to each well, and the plates were incubated for 1 h at 37°C. The plates were then transferred to a 65°C oven for 30 min (to destroy endogenous phosphatases), cooled, and 100 μL 5 mM p‐NPP in diethanolamine buffer was added to each well. The plates were read on a Dynatech MRX plate reader at 405 nM once the yellow color developed. Basal and response to a control maximum (10 μM isoprenaline) were measured in 6 wells per plate.

### 2.7 Data Analysis

#### 2.7.1 Whole Cell Binding

The affinity of ^3^H‐CGP12177 was determined from saturation binding by plotting the specific binding (SB, Equation 1) of ^3^H‐CGP12177 using the non‐linear regression program Prism 10 and Equation (1):

(1) $$\;\mathrm{SB}=\;\frac{\left(A\times B\max\right)}{\left(A+K_{D}\right)}$$

where *A* is the concentration of ^3^H‐CGP12177, *B*max is the maximal specific binding, and *K*_D_ is the dissociation constant of ^3^H‐CGP12177.

Other ligands affinities were determined from competition binding, where a sigmoidal response curve was then fitted to the data and the IC_50_ (concentration required to inhibit 50% of the specific binding) determined using Equation (2).

(2) $$\%\text{uninhibited binding}=\;100\;–\frac{\left(100\times A\right)\;}{\left(A+{\mathrm{IC}}_{50}\right)}+\;\mathrm{NS}$$

where *A* is the concentration of the competing ligand, and NS is the non‐specific binding.

From the IC_50_ and known concentration of ^3^H‐CGP12177, a *K*_D_ value (concentration at which half the receptors are bound) for competing ligands was calculated using Equation (3) (Cheng–Prusoff equation):
(3)

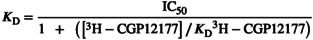




For relative assessment of the duration of binding, the rightward shift of the sigmoidal concentration response curve from the control and duration plate (normalized to total and non‐specific binding for each plate) was noted [23]. Shorter duration ligands, removed during the wash and/or dissociating from the receptor during the 2 h ^3^H‐CGP12177 incubation, would result in more ^3^H‐CGP12177 binding and a larger rightward shift of the concentration response curve, whereas longer‐acting ligands that did not dissociate (during wash or subsequent 2 h ^3^H‐CGP12177 incubation) would result in similar ^3^H‐CGP12177 binding as control.

#### 2.7.2 ^*3*^H‐cAMP Accumulation and CRE‐SPAP Responses

Agonist responses were best described by a one‐site sigmoidal concentration response curve (Equation 4):

(4) $$\text{Response}=\frac{E\max\times \left[A\right]}{{\mathrm{EC}}_{50}+\left[A\right]}$$

where *E*max is the maximum response, [*A*] is the agonist concentration, and EC_50_ is the concentration of agonist that produces 50% of the maximal response. Basal and 10 μM isoprenaline concentration responses allowed agonist responses to be expressed as a percentage of the maximum response to isoprenaline.

Antagonist affinities (*K*_D_ values) were calculated from the rightward parallel shift of the agonist concentration response curve measured in the absence and presence of a fixed concentration of antagonist in the same plate using the following (Gaddam equation):

$$\mathrm{DR}=1+\frac{\left[B\right]}{K_{D}}$$

where DR (dose ratio) is the ratio of the agonist concentration required to stimulate an identical response in the presence and absence of a fixed concentration of antagonist [*B*].

As many agonists appear as full or almost agonists in the functional assays. Potency (EC_50_) depends on both the affinity (ability to bind) and the intrinsic efficacy (ability to stimulate a response) of the compound. Intrinsic efficacy was expressed as an efficacy ratio (*K*_D_/EC_50_; [30, 32]).

Data for isoprenaline (less stable, COMT‐sensitive catecholamine that has an added complication of altered antagonist affinity measurements in prolonged assays, [33]) is given in the Supporting Information.

## 3 Results

### 3.1 Affinity of Ligands for CHO‐β_2_ and CHO‐β_1_— ^3^H‐CGP12177 Whole Cell Binding

**FIGURE 1:**
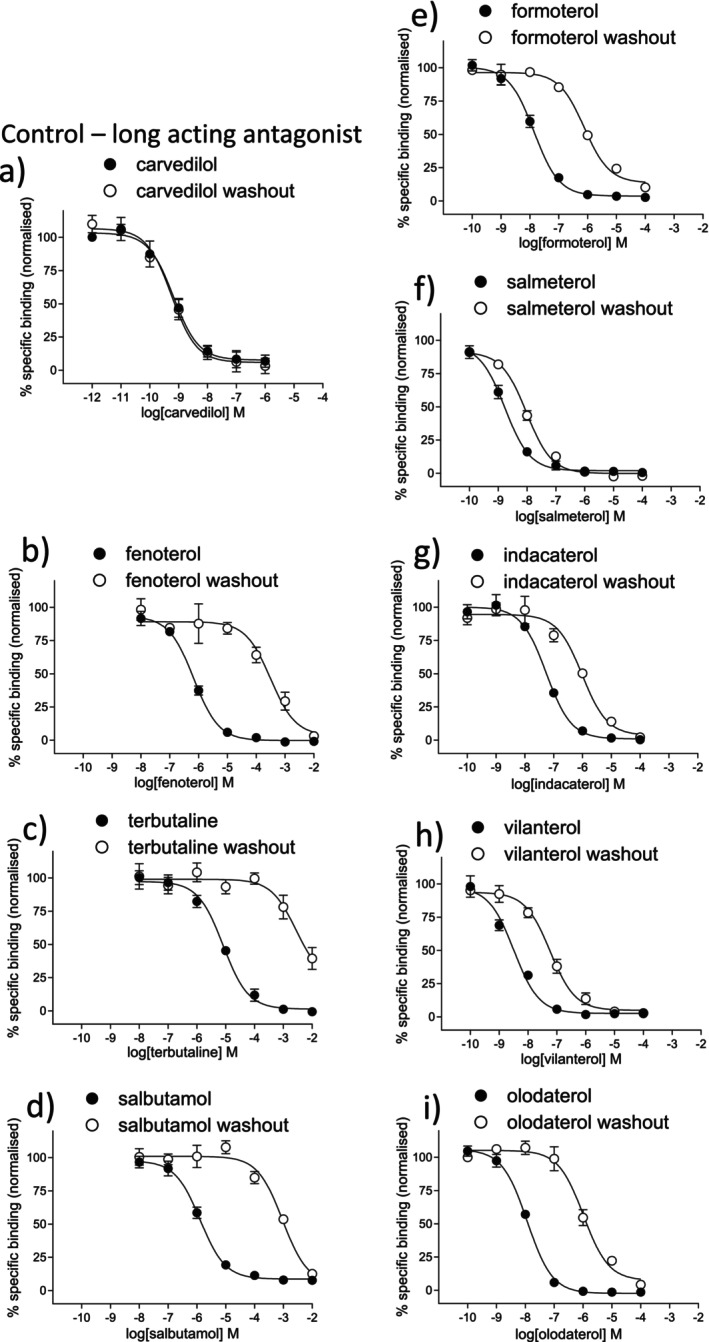
Inhibition of ^3^H‐CGP12177 specific binding in whole CHO‐β_2_ cells in response to (a) carvedilol, (b) fenoterol, (c) terbutaline, (d) salbutamol, (e) formoterol, (f) salmeterol, (g) indacaterol, (h) vilanterol, and (i) olodaterol and following washout. Non‐specific binding was determined by 10 μM propranolol and data points are mean ± SEM of triplicate determinations. The concentrations of ^3^H‐CGP12177 present in these experiments were (a) 0.77 nM, (b) 0.66 nM, (c) 0.68 nM, (d) 0.66 nM, (e) 0.83 nM, (f) 0.77 nM, (g) 0.47 nM, (h) 0.76 nM, and (i) 0.77 nM and these single experiments are representative of (a) 7, (b) 12, (c) 7, (d) 11, (e) 9, (f) 10, (g) 20, (h) 13, and (i) 10 separate experiments.

^3^H‐CGP12177 saturation binding yielded a *K*_D_ value of 0.16 ± 0.02 nM (*n* = 15) in the CHO‐β_2_ cells, similar to previous studies [29] and a receptor expression level of 116 ± 15 fmol/mg protein (Figure S1a). Propranolol had high affinity (p*K*_D_ 9.28, 0.5 nM Table 1, Figure S1b); ICI118551, a known β_2_‐selective antagonist, also had high affinity (p*K*_D_ 9.36, 0.4 nM), whilst the affinity of CGP20712A (β_1_‐selective antagonist) was low (p*K*_D_ 5.78, 1660 nM) as in previous studies [29]. Comparing this with CHO‐β_1_ data obtained in parallel experiments, ICI118551 had a 525‐fold higher affinity for the CHO‐β_2_ cell line, and CGP20712A had a 631‐fold higher affinity for the CHO‐β_1_ cell line, confirming the presence of the respective receptors. The affinities of the β_2_‐agonists were determined. Whilst the SABAs were low affinity, the affinity and selectivity of the LABAs and uLABAs varied considerably (Figure 1, Table 1).

**TABLE 1:** p*K*_D_ values, from ^3^H‐CGP12177 whole cell binding for ligands obtained in the CHO‐β_2_ and CHO‐β_1_ cells and the rightward log shift following washout of the ligand. Values are mean ± sem of *n* separate experiments. The log selectivity for the compounds for the β_2_ over the β_1_‐adrenoceptor is also given. Thus, fenoterol has a log 2.17 (148‐fold) higher affinity for the β_2_ than β_1_‐adrenoceptor and is readily washed out (i.e., short acting) with a washout shift of log 2.90 (794‐fold), whereas carvedilol is 1.24 (17‐fold) β_2_‐selective and is not washed out (i.e., long acting) with a washout shift of log 0.16 (1.4‐fold). CGP20712A (−2.80) is 631‐fold β_1_‐selective.

	CHO‐β_2_				CHO‐β_1_				Log β_2_‐selectivity
	pK_D_	*n*	Log shift	*n*	p*K*_D_	*n*	Log shift	*n*	
Fenoterol	6.87 ± 0.06	14	2.90 ± 0.05	12	4.70 ± 0.06	13	> 2	12	2.17
Salbutamol	6.26 ± 0.07	18	2.80 ± 0.05	11	4.64 ± 0.03	12	> 2	12	1.62
Terbutaline	5.59 ± 0.05	15	2.76 ± 0.11	7	3.73 ± 0.07	11	> 1.5	11	1.86
Formoterol	8.51 ± 0.05	13	2.16 ± 0.12	9	5.79 ± 0.04	11	> 2	11	2.72
Salmeterol	9.35 ± 0.05	14	0.95 ± 0.08	10	5.41 ± 0.05	12	1.21 ± 0.05	11	3.94
Indacaterol	7.90 ± 0.06	20	1.23 ± 0.07	20	6.30 ± 0.04	16	1.50 ± 0.04	16	1.60
Olodaterol	8.70 ± 0.08	10	1.91 ± 0.09	10	6.41 ± 0.03	9	> 2	9	2.29
Vilanterol	9.04 ± 0.06	13	1.17 ± 0.09	13	5.55 ± 0.04	11	1.38 ± 0.08	11	3.49
Carvedilol	9.94 ± 0.07	7	0.16 ± 0.07	7	8.70 ± 0.05	7	0.19 ± 0.02	7	1.24
ICI118551	9.36 ± 0.04	13			6.64 ± 0.08	6			2.72
Propranolol	9.28 ± 0.06	11			8.13 ± 0.08	7			1.15
CGP20712A	5.78 ± 0.05	8			8.58 ± 0.09	7			−2.80
CGP12177	9.61 ± 0.07	9			8.97 ± 0.06	7			0.64

### 3.2 Duration of Binding for CHO‐β_2_ and CHO‐β_1_

A measure of the longevity of binding was determined using a washout assay. Carvedilol (a long duration β‐antagonist, [23]) inhibited the binding of ^3^H‐CGP12177 even after washout with very little rightward shift of the binding curve (0.16 log units, 1.4‐fold Table 1, Figure 1). SABAs were readily washed out, resulting in a large rightward shift (log 2.76–2.90 rightward shift = 575–794‐fold). The duration of binding for LABAs and uLABAs varied considerably, with formoterol and olodaterol appearing to be shorter acting than salmeterol, vilanterol, or indacaterol.

### 3.3 Intrinsic Efficacy in CHO‐β_2_ and CHO‐β_1_— ^3^H‐cAMP Accumulation

**FIGURE 2:**
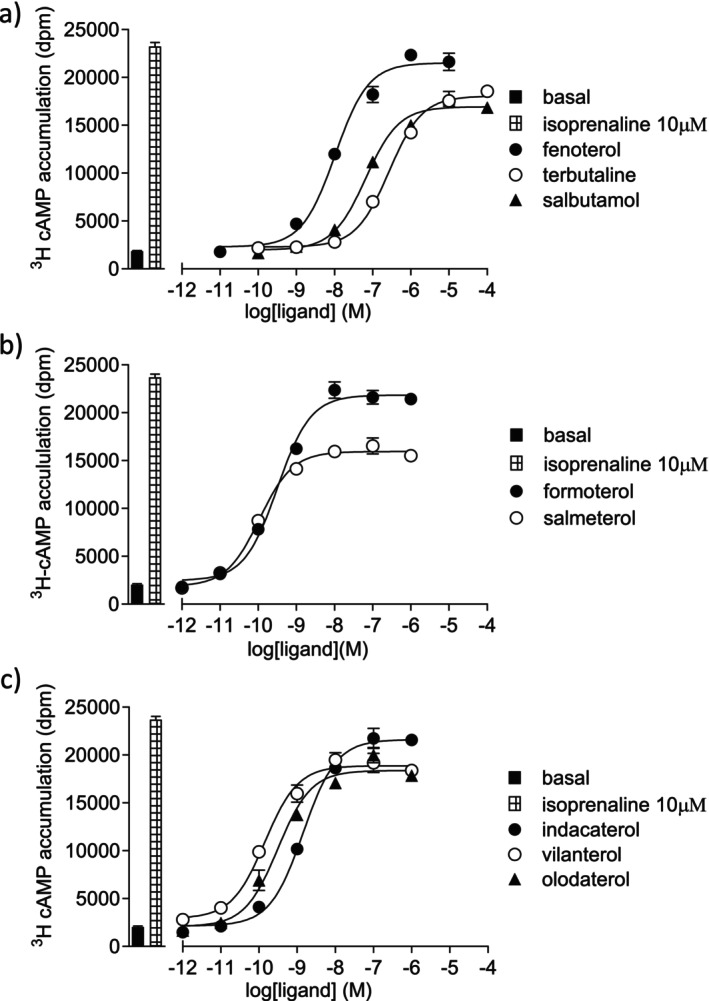
^3^H‐cAMP accumulation in CHO‐β_2_ cells in response to (a) SABAs, (b) LABAs, and (c) uLABAs. Bars represent basal and ^3^H‐cAMP accumulation in response to 10 μM isoprenaline. Data points are mean ± SEM of triplicate determinations and these single experiments are representative of seven separate experiments.

The ability of compounds to stimulate agonist responses was measured using the second messenger cAMP. All β_2_‐agonists stimulated an increase in ^3^H‐cAMP production, with formoterol stimulating the largest response (97%), relative to 10 μM isoprenaline (Figure 2, Table 2). Salmeterol was a partial agonist, stimulating the lowest overall response (63%). The potency (EC_50_) of the ligands varied considerably, but as this is dependent upon affinity, the efficacy ratio (*K*_D_/EC_50_) was calculated to isolate intrinsic efficacy. Fenoterol had the highest intrinsic efficacy, with formoterol being the most efficacious LABA and indacaterol the most efficacious uLABA (Table 2). In the CHO‐β_1_ cells, fenoterol was also the most efficacious compound, followed by formoterol.

**TABLE 2:** pEC_50_ values and % response of that to 10 μM isoprenaline obtained from ^3^H‐cAMP accumulation in CHO‐β_2_ and CHO‐β_1_ cells. Values are mean ± SEM of *n* separate experiments. Log values of the efficacy ratio (ER, p*K*_D_/pEC_50_) is also given (where p*K*_D_ is taken from Table 1), thus fenoterol is the most efficacious compound (log ER 1.28 or 19‐fold), whereas salmeterol is the least efficacious compound (0.67, or 4.2‐fold).

	CHO‐β_2_				CHO‐β_1_			
	pEC_50_	% isop	*n*	Log ER	pEC_50_	% isop	*n*	Log ER
Fenoterol	8.15 ± 0.05	88.2 ± 2.3	7	1.28	8.47 ± 0.04	124.1 ± 2.9	7	3.77
Salbutamol	7.34 ± 0.09	78.3 ± 3.3	7	1.08	7.28 ± 0.05	127.6 ± 3.3	7	2.64
Terbutaline	6.63 ± 0.06	79.1 ± 2.7	7	1.04	6.62 ± 0.04	132.4 ± 4.8	7	2.89
Formoterol	9.54 ± 0.07	96.6 ± 1.8	7	1.03	9.19 ± 0.04	133.2 ± 4.1	7	3.40
Salmeterol	10.02 ± 0.07	62.9 ± 1.9	7	0.67	7.77 ± 0.06	131.3 ± 3.3	6	2.36
Indacaterol	8.85 ± 0.05	87.2 ± 2.1	7	0.95	9.01 ± 0.07	132.6 ± 2.1	7	2.71
Olodaterol	9.42 ± 0.07	79.7 ± 2.3	7	0.72	8.60 ± 0.04	138.0 ± 4.9	7	2.19
Vilanterol	9.92 ± 0.06	76.6 ± 1.8	7	0.88	7.94 ± 0.03	120.7 ± 3.7	7	2.39

### 3.4 Gene Transcription in CHO‐β_2_ and CHO‐β_1_—CRE‐SPAP Production

**FIGURE 3:**
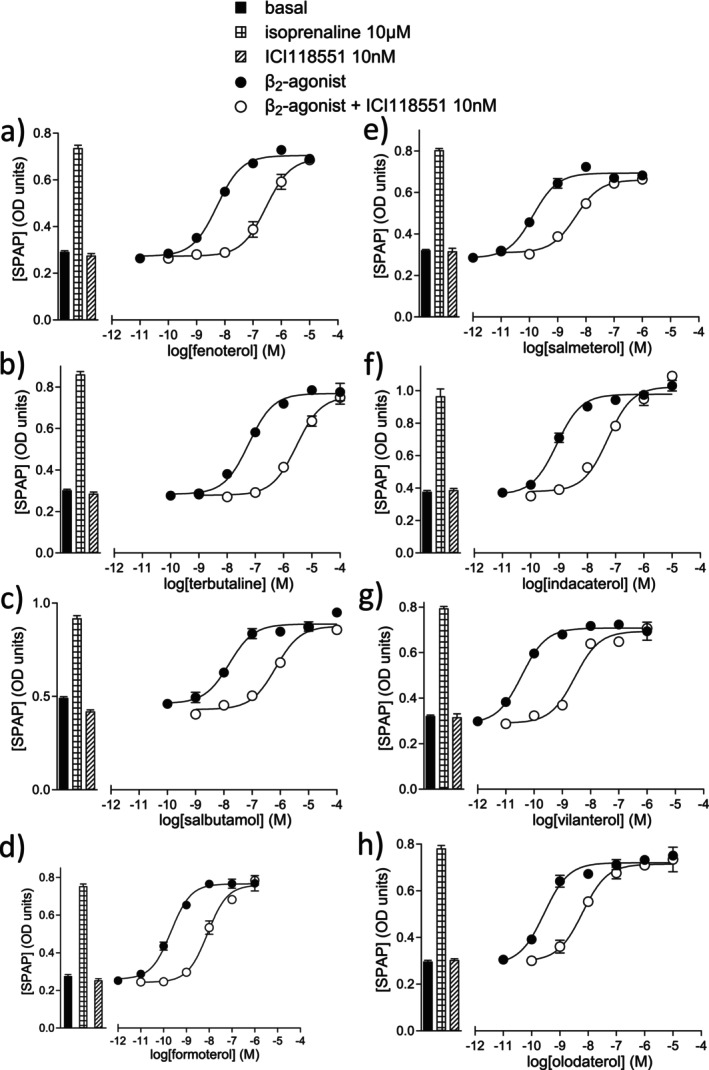
CRE‐SPAP production in CHO‐β_2_ cells in response to (a) fenoterol, (b) terbutaline, (c) salbutamol, (d) formoterol, (e) salmeterol, (f) indacaterol, (g) vilanterol, and (h) olodaterol in the absence and presence of 10 nM ICI118551. Bars represent basal and CRE‐SPAP production in response to 10 μM isoprenaline or 10 nM ICI118551 alone. Data points are mean ± SEM of triplicate determinations and these single experiments are representative of (a) 20, (b) 19, (c) 23, (d) 19, (e) 18, (f) 14, (g) 19, and (h) 21 separate experiments.

Down‐stream, longer‐term changes in gene transcription were also studied (Figure 3, Table 3). As with the cAMP measurements, salmeterol was the most partial β‐agonist at β_2_‐AR. The ability of antagonists to inhibit the responses was also assessed. ICI118551 and propranolol inhibited all agonist responses with high affinity, confirming they were β_2_‐mediated. In CHO‐β_1_ cells, fenoterol was again the most efficacious ligand, and the affinity of ICI118551 and propranolol was about 500 and 10‐fold less, respectively, as expected from the binding data.

**TABLE 3:** pEC_50_ values and % response of that to 10 μM isoprenaline obtained from CRE‐SPAP production in CHO‐β_2_ and CHO‐β_1_ cells, and p*K*_D_ values for ICI118551 and propranolol measured from a rightward parallel shift of the different agonists. Values are mean ± SEM of *n* separate experiments. Log values of the efficacy ratio (ER, pK_D_/pEC_50_) are given (p*K*_D_ taken from Table 1).

	pEC_50_	% isop	*n*	Log ER	pK_D_ ICI118551	*n*	p*K*_D_ propranolol	*n*
*CHO‐β*_*2*_								
Fenoterol	8.39 ± 0.08	91.1 ± 1.9	20	1.52	9.69 ± 0.06	20		
Salbutamol	7.53 ± 0.07	87.1 ± 1.6	23	1.27	9.77 ± 0.03	40	9.57 ± 0.05	31
Terbutaline	7.13 ± 0.09	86.6 ± 2.8	20	1.54	9.82 ± 0.08	19		
Formoterol	9.77 ± 0.06	93.0 ± 2.7	19	1.26	9.83 ± 0.06	21		
Salmeterol	10.07 ± 0.05	70.4 ± 3.3	19	0.73	9.73 ± 0.09	18		
Indacaterol	9.26 ± 0.08	101.3 ± 2.1	15	1.36	9.85 ± 0.09	14		
Olodaterol	9.38 ± 0.11	90.1 ± 1.9	21	0.68	9.58 ± 0.07	24	9.68 ± 0.11	10
Vilanterol	10.32 ± 0.05	85.3 ± 1.9	21	1.28	9.75 ± 0.07	19		
*CHO‐β*_*1*_								
Fenoterol	7.93 ± 0.09	83.6 ± 1.9	19	3.23	7.09 ± 0.05	19		
Salbutamol	7.00 ± 0.14	81.3 ± 3.8	18	2.36	7.22 ± 0.03	22	8.82 ± 0.06	10
Terbutaline	6.60 ± 0.08	76.9 ± 2.9	20	2.87	7.20 ± 0.05	19		
Formoterol	8.73 ± 0.09	98.7 ± 2.9	20	2.94	7.15 ± 0.06	19		
Salmeterol	7.17 ± 0.07	93.6 ± 2.3	18	1.76	6.94 ± 0.04	17		
Indacaterol	8.92 ± 0.08	96.7 ± 4.6	17	2.62	6.99 ± 0.07	15		
Olodaterol	8.21 ± 0.12	81.7 ± 3.4	24	1.80	6.94 ± 0.07	27	8.76 ± 0.09	11
Vilanterol	7.87 ± 0.09	76.9 ± 3.5	19	2.32	7.05 ± 0.04	17		

### 3.5 Investigation of Affinity and Efficacy at the β_2_‐Polymorphic Variants

Initial in vitro studies suggested that naturally occurring β_2_‐adrenoceptor polymorphisms may affect molecular pharmacological properties [28]. Subsequent clinical trials have been mixed, with some suggesting polymorphisms are important (e.g., [34, 35]), while others do not (e.g., [36, 37, 38, 39, 40]). There is very little data for uLABAs. The effects of the four polymorphisms were therefore investigated. While the affinity of ^3^H‐CGP12177 and propranolol remained similar for all polymorphic variants (Table S1), the affinity for ICI118551 was about sevenfold less for the β_2_‐ile164 receptor, in keeping with previous observations [27]. The affinity and duration of binding of the β_2_‐agonists were very similar in the polymorphic variants as for β_2_‐WT (Table S1 and Figure S2).

CRE‐SPAP gene transcription responses in the β_2_‐polymorphic receptors were again all similar to β_2_‐WT. Of the LABAs and uLABAs, formoterol and indacaterol remained the most efficacious and salmeterol and olodaterol the least efficacious, as in CHO‐β_2_‐WT. Again, all β_2_‐agonist responses were inhibited by ICI118551 and propranolol, yielding log K_D_ values similar to those of the β_2_‐WT. As expected from ^3^H‐CGP12177 binding, the affinity of ICI118551 was lower in the CHO‐β_2_‐ile164 cell line (Table S2 and Figure S3).

### 3.6 Investigation of Exosite β_2_‐H296K‐K305D

**FIGURE 4:**
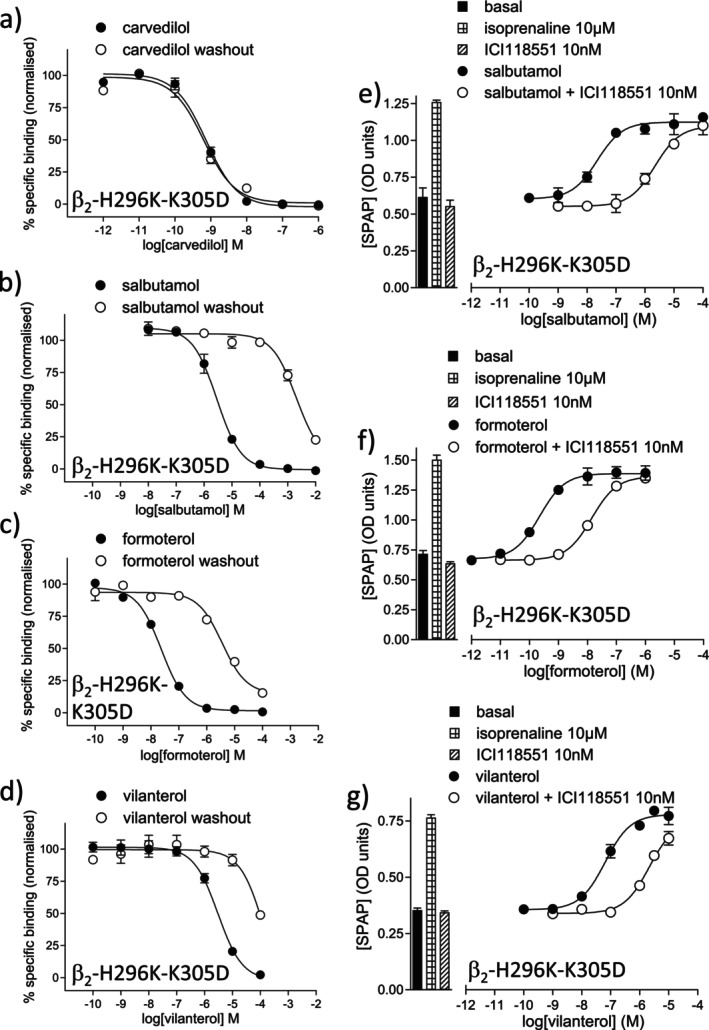
(a–d) Inhibition of ^3^H‐CGP12177 specific binding in whole cells stably expressing the double mutant CHO‐β_2_‐H296K‐K305D receptor in response to (a) carvedilol, (b) salbutamol, (c) formoterol, and (d) vilanterol and following washout. Non‐specific binding was determined by 10 μM propranolol and data points are mean ± SEM of triplicate determinations. The concentrations of ^3^H‐CGP12177 present in these experiments were (a) 0.52 nM, (b) 0.57 nM, (c) 0.67 nM, and (d) 0.57 nM and these single experiments are representative of (a) 6, (b) 9, (c) 9, and (d) 11 separate experiments. (e–g) CRE‐SPAP production in CHO‐β_2_‐H296K‐K305D cells in response to (e) salbutamol, (f) formoterol, and (g) vilanterol in the absence and presence of 10 nM ICI118551. Bars represent basal and CRE‐SPAP production in response to 10 μM isoprenaline or 10 nM ICI118551 alone. Data points are mean ± SEM of triplicate determinations, and these single experiments are representative of (e) 10, (f) 13, and (g) 10 separate experiments.

The alkyloxyphenyl side chain of salmeterol binds to a unique β_2_‐exosite involving a TM6 Histidine (H) at position 296 and an EL3 loop lysine (K) at position 305. Mutation of these two residues to their β_1_‐AR equivalent (lysine (K) and aspartate (D) respectively) abolishes this interaction whilst leaving other compound responses unchanged [23, 24]. The affinity of ^3^H‐CGP12177 in the CHO‐β_2_‐H296K‐K305D cells was 0.13 ± 0.02 nM (*n* = 5; 332 ± 140 fmol/mg protein), similar to β_2_‐WT. The affinity of the other ligands was also similar, except salmeterol and vilanterol, which were drastically reduced (Figure 4, Table 4).

**TABLE 4:** p*K*_D_ values from ^3^H‐CGP12177 whole cell binding for ligands obtained from CHO‐β_2_‐H296K‐K305D cells and the rightward log shift following washout of the ligands, as in Table 1. Values are mean ± SEM of *n* separate experiments. The β_2_‐WT data p*K*_D_ values and log shift from Table 1 is given. Thus, the affinity of fenoterol is log 0.27 (1.9‐fold) less at the β_2_‐H296K‐K305D compared to the β_2_‐WT, whereas the affinity of salmeterol and vilanterol is log 2.79 (617‐fold) and log 2.87 (741‐fold) less at β_2_‐H296K‐K305D compared to β_2_‐WT, respectively.

	CHO‐β_2_‐WT		CHO‐β_2_‐H296K‐K305D				Reduction in p*K*_D_ comparing CHO‐β_2_‐H296K‐K305D versus β_2_‐WT
	p*K*_D_	Log shift	p*K*_D_	*n*	Log shift	*n*	
Fenoterol	6.87	2.90	6.60 ± 0.05	10	2.95 ± 0.06	10	0.27
Salbutamol	6.26	2.80	6.21 ± 0.06	9	2.71 ± 0.10	9	0.05
Terbutaline	5.59	2.76	5.50 ± 0.03	12	2.97 ± 0.07	6	0.09
Formoterol	8.51	2.16	8.25 ± 0.08	9	2.24 ± 0.13	9	0.26
Salmeterol	9.35	0.95	6.56 ± 0.05^a^	9	1.30 ± 0.08	9	2.79
Indacaterol	7.90	1.23	7.58 ± 0.04	12	1.31 ± 0.08	12	0.32
Olodaterol	8.70	1.91	8.68 ± 0.06	8	2.15 ± 0.14	8	0.02
Vilanterol	9.04	1.17	6.17 ± 0.05^a^	12	1.54 ± 0.09	11	2.87
Carvedilol	9.94	0.16	9.81 ± 0.04	6	0.02 ± 0.06	6	0.13
ICI118551	9.36		9.49 ± 0.01	6			−0.13
Propranolol	9.28		9.23 ± 0.08	6			0.05
CGP20712A	5.78		5.83 ± 0.06	6			−0.05
CGP12177	9.61		9.59 ± 0.06	5			0.02

^a^ *p* < 0.0001 unpaired *t*‐test comparing p*K*_D_ values obtained in CHO‐β_2_‐WT with those obtained from the CHO‐β_2_‐H296K‐K305D. Thus the p*K*_D_ for salmeterol and vilanterol is different with *p* < 0.0001.

In the CRE‐SPAP functional assay, all β_2_‐agonists stimulated agonist responses in the CHO‐β_2_‐H296K‐K305D that were inhibited by ICI118551 and propranolol with high affinity; however, the agonist responses to salmeterol and vilanterol were considerably right‐shifted (Figure 4, Table 5). The intrinsic efficacies (efficacy ratio, Table 5) were similar to that of the β_2_‐WT. Thus, salmeterol and vilanterol have greatly reduced affinity for the β_2_‐H296K‐K305D receptor, which, as expected, reduced their potency, but once bound to the receptor, their intrinsic activity was unchanged.

**TABLE 5:** pEC_50_ values and % response of that to 10 μM isoprenaline obtained from CRE‐SPAP production in CHO‐β_2_‐H296K‐K305D cells and p*K*_D_ values for ICI118551 and propranolol measured from a rightward parallel shift of the different agonists. Values are mean ± SEM of *n* separate experiments. Log values of the efficacy ratio (ER, p*K*_D_/pEC_50_) are given (p*K*_D_ taken from Table 4). In addition, the change in pEC_50_ compared to the β_2_‐WT (from Table 3) is given. Thus, the potency (pEC_50_) of fenoterol is log 0.08 (1.2‐fold) less at the β_2_‐H296K‐K305D compared to the β_2_‐WT, whereas the potency of salmeterol and vilanterol are log 2.62 (417‐fold) and log 3.00 (1000‐fold) less respectively. Thus, the loss in potency of both salmeterol and vilanterol (Table 5) is explained by the loss in affinity (Table 4).

	CHO‐β_2_‐H296K‐K305D								Reduction in pEC_50_ comparing CHO‐β_2_‐H296K‐K305D versus β_2_‐WT
	pEC_50_	% isop	*n*	Log ER	p*K*_D_ ICI118551	*n*	p*K*_D_ propranolol	*n*	
Fenoterol	8.31 ± 0.09	93.9 ± 3.8	20	1.71	9.85 ± 0.09	17			0.08
Salbutamol	7.63 ± 0.12	81.6 ± 3.3	10	1.42	10.05 ± 0.06	15	9.79 ± 0.06	11	−0.10
Terbutaline	6.99 ± 0.08	82.7 ± 3.3	12	1.49	9.87 ± 0.06	11			0.23
Formoterol	9.86 ± 0.07	91.0 ± 2.8	14	1.61	9.92 ± 0.10	13			−0.09
Salmeterol	7.45 ± 0.07	87.9 ± 2.9	14	0.89	9.64 ± 0.05	13			2.62
Indacaterol	9.30 ± 0.06	96.4 ± 2.4	13	1.72	9.91 ± 0.08	9			−0.04
Olodaterol	9.48 ± 0.12	85.7 ± 2.6	16	0.80	9.61 ± 0.08	16	9.56 ± 0.10	10	−0.10
Vilanterol	7.32 ± 0.10	82.5 ± 3.5	11	1.15	9.72 ± 0.07	10			3.00

### 3.7 Investigation of Isoprenaline Responses

Isoprenaline and adrenaline (less stable, COMT‐sensitive catecholamines) are highly efficacious SABAs [30], their presence in β_2_‐CRE‐gene transcription assays has previously been noted to alter antagonist affinity measurements made from parallel shifts of agonist responses when compared to the presence of salbutamol or terbutaline [33]. Data for isoprenaline is given in Tables S3, S4 and Figure S4 and confirms that isoprenaline is a short‐acting low‐affinity agonist and, whilst the reason is unknown, the difference in antagonist affinity measurements when isoprenaline is present, compared to all the other β_2_‐agonists, remains so for β_2_‐WT and across all β_2_‐polymorphisms.

## 4 Discussion

There have been two recent changes regarding β_2_‐agonists: uLABAs for COPD (but not asthma), and asthma international guidelines where single MART formoterol + ICS inhalers are replacing separate SABA and ICS inhalers. However, as certain SABAs were linked to asthma deaths, LABAs are subject to FDA black box monotherapy warnings and uLABAs are licensed for COPD but not asthma, it is important to compare the β_2_‐agonist molecular pharmacological properties, including affinity (ability to bind to a receptor), intrinsic efficacy (ability to elicit a response) and duration of action.

As expected, SABAs have low affinity (micromolar) whereas LABAs and uLABAs have substantially higher affinity (0.4 nM for salmeterol, 13 nM for indacaterol) however, there was a large variation in β_2_ versus β_1_‐selectivity (Table 1). Indacaterol had only 40‐fold higher affinity for the β_2_‐adrenoceptor (similar to salbutamol) whereas salmeterol and vilanterol were 3090 and 8511‐fold selective, as previously observed [17, 19, 30].

Fenoterol (SABA, no longer clinically used) was the most efficacious β_1_‐AR agonist as well as being highly β_2_‐efficacious (Table 2). Previous studies suggest the intrinsic efficacy of fenoterol (e.g., cells [30]; tracheas [41, 42]; and heart rate increase following MDI inhalation [43]) can be similar to that of catecholamines. Direct comparisons demonstrate that salbutamol is a partial agonist compared to fenoterol (e.g., cells [30]; tracheas [44]; and following nebulizer or MDI inhalation [45, 46]). The efficacy order of isoprenaline > fenoterol > formoterol > salbutamol > salmeterol is well established ([1] and references therein). Although the cause for the previous asthma deaths remains unknown and contested, given their sudden nature, β_1_‐mediated tachyarrhythmias from readily systemically absorbed, highly efficacious agonists (with or without β_2_‐mediated chronotropic effects) were possible. Formoterol was the most efficacious LABA, and indacaterol the most efficacious uLABA (as in e.g., [19, 41, 47, 48]), and neither has been linked to asthma deaths. Salmeterol and olodaterol were consistently the least efficacious β_2_‐agonists.

Given the structural similarities between salmeterol and vilanterol, it is not surprising that they utilize the same exosite ([23, 24]; Tables 4 and 5). The hydrocarbon side‐chain interaction with the TM6‐EL3 exosite (H296‐K305) is vital for their very high affinity and β_2_‐selectivity. Mutating these two residues reduces the affinity to that of their head group, salbutamol. This exosite is not important for intrinsic efficacy—the vastly reduced potency (EC_50_) of salmeterol and vilanterol is explained by reduced affinity.

As expected, SABAs were short duration ligands, readily washed out with large rightward binding curve shifts (Table 1). The duration of LABAs and uLABAs was very variable with complete overlap of the two categories. Salmeterol and vilanterol were longer acting, whilst formoterol and olodaterol were more readily washed out. Of note, no β_2_‐agonist was as long acting as the antagonist carvedilol, suggesting there is potential to develop yet longer acting β_2_‐agonists.

There were no major differences in the affinity, intrinsic efficacy, or duration of the β_2_‐agonists at the β_2_‐polymorphisms compared to β_2_‐WT (Supporting Information). Despite mixed in vitro and asthma and COPD clinical results (e.g., [28, 34, 35, 36, 37, 38, 39, 40]), genome‐wide association studies have not linked polymorphisms with clinical traits [49]. Whilst there are very few studies examining uLABAs and β_2_‐polymorphisms, the lack of differences found here is similar to previous studies [26, 50, 51]. The β_2_‐ile164 variant has lower affinity for ICI118551; thus, it remains important to investigate drug action in naturally occurring polymorphisms.

Two theories exist regarding duration. The “exosite theory” involves drugs anchored to an additional specific exosite, separate from the orthosteric binding site, to increase duration at the receptor level. Salmeterol and vilanterol utilize an exosite for affinity. The “diffusion microkinetic theory” suggests higher lipophilicity drugs deposit in the cell membrane forming a reservoir to slowly leach out and bind to receptors [52, 53, 54, 55]. In this case, duration should be concentration dependent, with higher (supramaximal) doses resulting in larger reservoir deposition, giving longer lasting responses. This would be particularly effective for ligands delivered at higher local concentrations (e.g., inhalation) and could explain discrepancies between shorter and longer durations of formoterol, at equipotent and supramaximal concentrations, across different studies [56]. Indacaterol was designed with lipophilicity to enhance duration [54]. Salmeterol and vilanterol are also a highly lipophilic [14, 53].

Many in vitro studies have found formoterol to have a relatively short duration. Formoterol had a similar duration to fenoterol, salbutamol, and terbutaline in isolated human bronchus, whereas that for salmeterol was 40 times longer [57]. Histamine release from human lung (mast cells) following salbutamol was reduced at 2 h and lost by 4 h, formoterol reduced by 4 h and lost by 8 h, whereas salmeterol responses were still 75% maximal at 20 h [56]. Equipotent nebulized agents demonstrated that bronchodilation duration in conscious guinea pigs was less than 1.5 h for salbutamol, 3 h for formoterol, but no reduction with salmeterol at 6 h [56]. Other guinea pig tracheal studies also showed a short duration for formoterol (1.2 h, compared to 0.9 h for salbutamol and over 12 h for both salmeterol and indacaterol; [58]). In human trachea, formoterol duration of action (34 min) was 4.5 times longer than salbutamol (7.6 min), but salmeterol was longer (overall > 125 min; [41]). The finding of longer durations for formoterol was only apparent following serendipitous human inhalation studies [6, 16].

**FIGURE 5:**
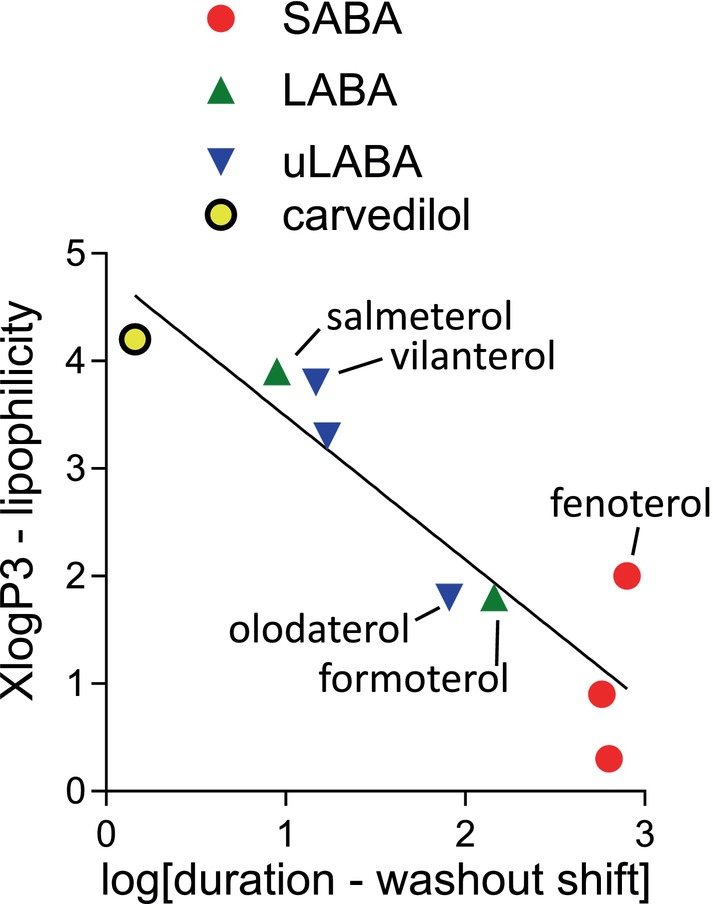
Correlation plot of duration of action at β_2_ (*x*‐axis, measured as the rightward shift in washout assays, Table 1) and lipophilicity (*y*‐axis, XlogP taken from PubChem https://pubchem.ncbi.nlm.nih.gov/docs/ March 28, 2025). The line is that of best fit through all compounds.

Here, the LABA formoterol and uLABA olodaterol appeared shorter in duration than their classification would suggest. Washout would not discriminate between removing ligand from membrane reservoirs versus removing ligand from exosites. There was, however, a strong correlation between duration (rightward shift following washout, Table 1) and calculated logP values (measure of lipophilicity, Figure 5). For salmeterol and vilanterol, β_2_‐H296K‐K305D removes their specific exosite, but the increase in washout rightward shift is marginal (log 0.35–0.37 or 2.8‐fold change compared β_2_‐WT, although greater than the other ligands) and yet these ligands remain longer acting at β_2_‐H296K‐K305D. Thus, although the exosite is responsible for their high affinity and selectivity, their lipophilicity may be more important for their long duration of action [53].

Clinical studies have suggested the superiority of MART formoterol + ICS inhalers for asthma [21]. Formoterol may be superior to other β_2_‐agonists if it possesses the optimal properties of β_2_‐selectivity, high but not too high efficacy, enough aqueous solubility for fast onset of action, but lipophilic enough to achieve a substantial membrane deposition for longer duration and little systemic absorption. However, other factors affect clinical outcomes. Many different inhaler devices exist that potentially affect particle size and deposition site (mouth to alveolus), speed of onset (solution vs. dry powder) as well as patient preference and ability to use. Whereas in vitro studies use compounds dissolved in aqueous solution, added at time zero, for clinical use, drugs arrive at the mucociliary surface, dissolve and diffuse through to the underlying epithelial and smooth muscle cells. Solubility, diffusion, and retention times of the different β_2_‐agonists in the different mucous and extracellular space environments (including if compounds are trapped/more concentrated in one of these environments effecting reservoir accumulation and/or receptor rebinding [59]) and proportion of membrane deposition vs. receptor binding [55] will all affect clinical outcomes. Likewise, changeable patient factors, e.g., speed of mucociliary clearance, volume of mucous, inflammation, and intercurrent infection will all affect onset, concentration reached, and retention of drug beyond any molecular β_2_‐pharmacological properties. Cost, device, environmental concerns (MDIs have higher greenhouse gas emissions than DPI; [60]) and availability of dual (u)LABA+ICS and triple (u)LABA+LAMA+ICS combination inhalers determine specific β_2_‐agonist drug availability. Thus, the individual molecular pharmacological properties of the different β_2_‐agonists only form a small part of the clinical outcomes in patients.

In conclusion, there is a wide range in the affinity, selectivity, intrinsic efficacy and duration of β_2_‐agonists. Whilst the properties of SABAs are reasonably similar, the molecular pharmacological properties of the LABAs and uLABA vary so much the two classes overlap. Salmeterol and vilanterol's exosite interaction explains their very high affinity and selectivity (intrinsic efficacy unaffected), but lipophilicity appears more important for duration. However, non‐pharmacological properties (including physicochemical, device preference and combination availability) maybe as, if not more important in overall clinical outcomes given the rather varied molecular pharmacological properties of the β_2_‐agonists themselves. From a pharmacological perspective, as salmeterol (“uLABA” in vitro profile), and formoterol (high efficacy) are both safe in asthma long‐term, uLABA+ICS combinations may be safe if given inadvertently in asthma rather than COPD.

## Author Contributions

**Richard G. W. Proudman:** data curation, methodology. **Jillian G. Baker:** conceptualization, investigation, funding acquisition, writing – original draft, writing – review and editing, methodology, formal analysis, project administration, data curation, validation.

## Conflicts of Interest

The authors declare no conflicts of interest.

## Supporting information


Data S1.

## Data Availability

The data that support the findings of this study are available from the corresponding author upon reasonable request.
