# Supplementary material for: A Comparison of the Molecular Pharmacological Properties of Current Short, Long, and Ultra‐Long‐Acting β2‐Agonists Used for Asthma and COPD
Source: Pharmacol Res Perspect. 2025 Aug 31;13(5):e70154. doi: 10.1002/prp2.70154 (PMC12399788; doi:10.1002/prp2.70154)
Supplement: Supplementary file 1 — Data S1. [file PRP2-13-e70154-s001.docx]

**Supplementary Information: A comparison of the molecular pharmacological properties of current short, long and ultra-long-acting β_2_-agonists used for asthma and COPD.**

***Effect of the four naturally occurring polymorphic variants on the molecular pharmacological properties of β_2_-agonists***

The affinity, duration and intrinsic efficacy of the β_2_-agonists was assessed in CHO cell lines stably expressing each of the 4 naturally occurring polymorphisms. K_D_ values obtained from ^3^H-CGP12177 saturation binding were very similar in the polymorphic cell lines to that of β_2_-WT: 0.14 ± 0.1nM (n=5) for CHO-β_2_-gly16 (340 ± 32fmol/mg protein); 0.14 ± 0.1nM (n=7) for CHO-β_2_-gln27 (368 ± 36fmol/mg protein); 0.13 ± 0.01nM (n=9) for CHO-β_2_-met34 (134 ± 44fmol/mg protein); and 0.13 ± 0.01nM for CHO-β_2_ile164 (152 ± 36fmol/mg protein). As expected from previous findings (Baker et al., 2014), the affinity of ICI118551 was reduced for the β_2_-ile164 variant (both in binding studies Table S1 and from inhibition of the agonist responses, Table S2, Figure S2). The affinity, duration and intrinsic efficacy of the β_2_-agonists was found to be similar to that of β_2_-WT.

Supplementary Table S1

pK_D_ values, from ^3^H-CGP12177 whole cell binding for ligands obtained in cell lines expressing each of the β_2_-AR polymorphic receptors: CHO-β_2_-gly16, CHO-β_2_-gln27, CHO-β_2_-met34 and CHO-β_2_-ile164. The rightward log shift following washout of the ligands are also given. Values are mean ± sem of n separate experiments. The β_2_-WT data is given in Table 1 of the main manuscript.

|  | CHO-β_2_-WT | | CHO-β_2_-gly16 | | | | CHO-β_2_-gln27 | | | | CHO-β_2_-met34 | | | | CHO-β_2_-ile164 | | | |
| --- | --- | --- | --- | --- | --- | --- | --- | --- | --- | --- | --- | --- | --- | --- | --- | --- | --- | --- |
|  | pK_D_ | Log shift | pK_D_ | n | Log shift | n | pK_D_ | n | Log shift | n | pK_D_ | n | Log shift | n | pK_D_ | n | Log shift | n |
| Fenoterol | 6.87 | 2.90 | 6.76 ± 0.06 | 10 | 3.10 ± 0.07 | 10 | 6.74 ± 0.08 | 9 | 3.01 ± 0.09 | 9 | 6.97 ± 0.06 | 12 | 3.21 ± 0.06 | 12 | 6.51 ± 0.08 | 10 | 2.92 ± 0.06 | 8 |
| Salbutamol | 6.26 | 2.80 | -6.05 ± 0.06 | 12 | 2.85 ± 0.09 | 8 | 6.08 ± 0.05 | 11 | 2.70 ± 0.10 | 9 | 6.10 ± 0.08 | 15 | 2.93 ± 0.10 | 6 | 5.91 ± 0.06 | 13 | 2.73 ± 0.12 | 8 |
| Terbutaline | 5.59 | 2.76 | 5.52 ± 0.05 | 12 | 2.94 ± 0.06 | 6 | 5.59 ± 0.03 | 13 | 3.14 ± 0.09 | 6 | 5.59 ± 0.06 | 16 | 2.80 ± 0.12 | 6 | 5.24 ± 0.05** | 14 | 2.98 ± 0.05 | 6 |
|  |  |  |  |  |  |  |  |  |  |  |  |  |  |  |  |  |  |  |
| Formoterol | 8.51 | 2.16 | 8.27 ± 0.07 | 12 | 2.53 ± 0.11 | 9 | 8.34 ± 0.08 | 11 | 2.46 ± 0.17 | 8 | 8.42 ± 0.06 | 12 | 2.17 ± 0.10 | 9 | 8.08 ± 0.03 | 10 | 2.38 ± 0.09 | 8 |
| Salmeterol | 9.35 | 0.95 | 9.30 ± 0.07 | 10 | 1.12 ± 0.13 | 8 | 9.24 ± 0.08 | 10 | 0.92 ± 0.08 | 7 | 9.17 ± 0.11 | 11 | 0.66 ± 0.05 | 7 | 9.07 ± 0.10 | 9 | 0.76 ± 0.09 | 7 |
|  |  |  |  |  |  |  |  |  |  |  |  |  |  |  |  |  |  |  |
| Indacaterol | 7.90 | 1.23 | 7.78 ± 0.05 | 11 | 1.20 ± 0.06 | 11 | 7.77 ± 0.03 | 11 | 1.21 ± 0.09 | 11 | 7.95 ± 0.04 | 14 | 1.27 ± 0.08 | 14 | 7.55 ± 0.04** | 11 | 1.15 ± 0.10 | 11 |
| Olodaterol | 8.70 | 1.91 | 8.75 ± 0.09 | 8 | 2.18 ± 0.06 | 8 | 8.73 ± 0.06 | 8 | 2.21 ± 0.08 | 8 | 8.91 ± 0.04 | 10 | 2.24 ± 0.06 | 10 | 8.48 ± 0.11 | 9 | 2.06 ± 0.12 | 8 |
| Vilanterol | 9.04 | 1.17 | 9.01 ± 0.05 | 10 | 1.25 ± 0.07 | 10 | 9.00 ± 0.07 | 10 | 1.25 ± 0.09 | 10 | 9.16 ± 0.05 | 11 | 1.40 ± 0.11 | 11 | 8.78 ± 0.10 | 9 | 1.06 ± 0.12 | 9 |
|  |  |  |  |  |  |  |  |  |  |  |  |  |  |  |  |  |  |  |
| Carvedilol | 9.94 | 0.16 | 9.84 ± 0.04 | 6 | 0.08 ± 0.05 | 6 | 9.88 ± 0.06 | 6 | 0.10 ± 0.07 | 6 | 10.10 ± 0.08 | 7 | 0.03 ± 0.04 | 7 | 9.79 ± 0.05 | 5 | 0.26 ± 0.09 | 5 |
| ICI118551 | 9.36 |  | 9.33 ± 0.04 | 8 |  |  | 9.33 ± 0.04 | 8 |  |  | 9.41 ± 0.04 | 11 |  |  | 8.52 ± 0.06** | 8 |  |  |
| Propranolol | 9.28 |  | 9.34 ± 0.06 | 6 |  |  | 9.31 ± 0.04 | 6 |  |  | 9.39 ± 0.05 | 9 |  |  | 9.18 ± 0.04 | 6 |  |  |
| CGP20712A | 5.78 |  | 5.89 ± 0.06 | 6 |  |  | 5.86 ± 0.03 | 6 |  |  | 5.83 ± 0.03 | 9 |  |  | 5.21 ± 0.05** | 6 |  |  |
| CGP12177 | 9.61 |  | 9.60 ± 0.05 | 7 |  |  | 9.61 ± 0.02 | 7 |  |  | 9.79 ± 0.09 | 9 |  |  | 9.65 ± 0.06 | 7 |  |  |

**p<0.0001 One-way ANOVA with post hoc Newman-Keuls comparing pK_D_ values obtained in all polymorphic variants with those obtained from the β_2_-WT. Thus e.g. the pK_D_ for ICI118551 in β_2_-ile164 is different from that obtained from the β_2_-WT with p<0.0001.

Supplementary Table S2

pEC_50_ values and % response of that to 10µM isoprenaline obtained from CRE-SPAP production in CHO-β_2_-gly16, CHO-β_2_-gln27, CHO-β_2_-met34 and CHO-β_2_ile164 cells and pK_D_ values for ICI118551 and propranolol measured from a rightward parallel shift of the different agonists. Values are mean ± sem of n separate experiments. Log values of the efficacy ratio (ER, pK_D_/pEC_50_) are given (pK_D_ taken from Supplementary Table S1). The β_2_-WT data is taken from Table 3 of the main manuscript.

|  | CHO-β_2_-WT | CHO-β_2_-gly16 | | | | | | | | CHO-β_2_-gln27 | | | | | | | |
| --- | --- | --- | --- | --- | --- | --- | --- | --- | --- | --- | --- | --- | --- | --- | --- | --- | --- |
|  | pEC_50_ | pEC_50_ | % isop | n | Log ER | pK_D_ ICI118551 | n | pK_D_ propranolol | n | pEC_50_ | % isop | n | Log ER | pK_D_ ICI118551 | n | pK_D_ propranolol | n |
| Fenoterol | 8.39 | 9.17 ± 0.07 | 95.3 ± 4.4 | 16 | 2.41 | 9.84 ± 0.08 | 15 |  |  | 9.26 ± 0.06 | 94.7 ± 2.9 | 19 | 2.52 | 9.96 ± 0.05 | 15 |  |  |
| Salbutamol | 7.53 | 7.93 ± 0.06 | 90.6 ± 3.2 | 16 | 1.88 | 9.87 ± 0.05 | 21 | 9.78 ± 0.08 | 21 | 8.04 ± 0.06 | 90.9 ± 2.6 | 15 | 1.96 | 9.83 ± 0.05 | 21 | 9.76 ± 0.05 | 20 |
| Terbutaline | 7.13 | 7.75 ± 0.12 | 88.3 ± 3.4 | 11 | 2.23 | 9.98 ± 0.06 | 10 |  |  | 7.80 ± 0.12 | 91.5 ± 2.5 | 10 | 2.21 | 9.90 ± 0.11 | 9 |  |  |
|  |  |  |  |  |  |  |  |  |  |  |  |  |  |  |  |  |  |
| Formoterol | 9.77 | 10.50 ± 0.12 | 95.8 ± 4.1 | 13 | 2.23 | 10.02 ± 0.07 | 12 |  |  | 10.56 ± 0.06 | 92.0 ± 3.5 | 12 | 2.22 | 10.04 ± 0.08 | 11 |  |  |
| Salmeterol | 10.07 | 10.33 ± 0.06 | 84.8 ± 3.7 | 10 | 1.03 | 9.55 ± 0.06 | 10 |  |  | 10.41 ± 0.06 | 90.3 ± 3.4 | 12 | 1.17 | 9.57 ± 0.05 | 11 |  |  |
|  |  |  |  |  |  |  |  |  |  |  |  |  |  |  |  |  |  |
| Indacaterol | 9.26 | 10.13 ± 0.16 | 92.8 ± 3.8 | 11 | 2.35 | 10.10 ± 0.08* | 15 |  |  | 10.18 ± 0.12 | 99.0 ± 3.2 | 12 | 2.41 | 9.98 ± 0.05 | 15 |  |  |
| Olodaterol | 9.38 | 9.96 ± 0.13 | 94.0 ± 3.0 | 13 | 1.21 | 9.62 ± 0.10 | 14 | 9.68 ± 0.12 | 8 | 10.18 ± 0.16 | 94.2 ± 2.7 | 12 | 1.45 | 9.69 ± 0.10 | 15 | 9.74 ± 0.12 | 9 |
| Vilanterol | 10.32 | 10.73 ± 0.07 | 85.8 ± 5.3 | 10 | 1.72 | 9.79 ± 0.09 | 9 |  |  | 10.83 ± 0.06 | 97.2 ± 3.3 | 9 | 1.83 | 9.81 ± 0.07 | 9 |  |  |

|  | CHO-β_2_-met34 | | | | | | | | CHO-β_2_-ile164 | | | | | | | |
| --- | --- | --- | --- | --- | --- | --- | --- | --- | --- | --- | --- | --- | --- | --- | --- | --- |
|  | pEC_50_ | % isop | n | Log ER | pK_D_ ICI118551 | n | pK_D_ propranolol | n | pEC_50_ | % isop | n | Log ER | pK_D_ ICI118551 | n | pK_D_ propranolol | n |
| Fenoterol | 8.75 ± 0.08 | 99.1 ± 3.7 | 16 | 1.78 | 9.86 ± 0.04 | 13 |  |  | 8.16 ± 0.12 | 93.0 ± 4.3 | 17 | 1.65 | 8.63 ± 0.04** | 16 |  |  |
| Salbutamol | 7.83 ± 0.10 | 95.9 ± 2.3 | 16 | 1.73 | 9.75 ± 0.06 | 24 | 9.74 ± 0.07 | 25 | 7.22 ± 0.08 | 79.3 ± 3.6 | 17 | 1.31 | 8.81 ± 0.06** | 23 | 9.46 ± 0.05 | 22 |
| Terbutaline | 7.43 ± 0.15 | 92.2 ± 4.4 | 11 | 1.84 | 9.83 ± 0.06 | 11 |  |  | 6.58 ± 0.09 | 82.6 ± 4.1 | 10 | 1.34 | 8.76 ± 0.03** | 9 |  |  |
|  |  |  |  |  |  |  |  |  |  |  |  |  |  |  |  |  |
| Formoterol | 10.14 ± 0.10 | 98.0 ± 4.3 | 10 | 1.72 | 9.77 ± 0.04 | 10 |  |  | 9.40 ± 0.14 | 87.7 ± 4.2 | 15 | 1.32 | 8.80 ± 0.07** | 14 |  |  |
| Salmeterol | 9.95 ± 0.09 | 83.8 ± 2.4 | 11 | 0.78 | 9.62 ± 0.05 | 10 |  |  | 9.76 ± 0.04 | 53.2 ± 2.7 | 13 | 0.69 | 8.72 ± 0.05** | 12 |  |  |
|  |  |  |  |  |  |  |  |  |  |  |  |  |  |  |  |  |
| Indacaterol | 9.35 ± 0.06 | 97.0 ± 2.0 | 20 | 1.40 | 9.67 ± 0.04 | 16 |  |  | 9.21 ± 0.05 | 94.1 ± 3.1 | 12 | 1.66 | 8.49 ± 0.12** | 16 |  |  |
| Olodaterol | 9.80 ± 0.14 | 95.8 ± 2.4 | 14 | 0.89 | 9.68 ± 0.05 | 15 | 9.61 ± 0.06 | 12 | 9.17 ± 0.10 | 79.4 ± 4.9 | 14 | 0.69 | 8.58 ± 0.13** | 15 | 9.33 ± 0.15 | 10 |
| Vilanterol | 10.53 ± 0.07 | 91.5 ± 2.7 | 11 | 1.37 | 9.71 ± 0.07 | 10 |  |  | 9.94 ± 0.09 | 75.6 ± 5.5 | 12 | 1.16 | 8.69 ± 0.07** | 11 |  |  |

**p<0.0001 One-way ANOVA with post hoc Newman-Keuls comparing pK_D_ values for ICI118551 with those obtained in the presence of the same agonist in all polymorphic variants with those obtained from the β_2_-WT (Table 3). Thus e.g. the pK_D_ for ICI118551 in the presence of fenoterol in β_2_-ile164 is different from that obtained in the presence of fenoterol from the β_2_-WT with p<0.0001. The pK_D_ values for propranolol were not different.

*p<0.05 One-way ANOVA with post hoc Newman-Keuls comparing pK_D_ values for ICI118551 with those obtained in the presence of the same agonist in all polymorphic variants with those obtained from the β_2_-WT (Table 3). Thus the pK_D_ for ICI118551 in the presence of indacaterol in β_2_-gly16 is different from that obtained from the β_2_-WT with p<0.05.

***The affinity, duration of action and functional responses to isoprenaline***

The catecholamines (isoprenaline, adrenaline and noradrenaline) have previously been studied in CHO cells stably expressing the human β_1_ and β_2_-adrenoceptors, including those expressing CRE-SPAP reporter genes, at binding, cAMP and CRE-SPAP gene transcription and over multiple time points (e.g. Baker et al., 2003 and 2004; Baker, 2005 and 2010). As expected, they are highly efficacious agonists (at β_1_ and β_2_). Isoprenaline and adrenaline have previously been used as inhaled SABA for asthma although were associated with asthma deaths (similar to fenoterol and potentially orciprenaline, Crompton, 2006; Pearce, 2007, Baker and Shaw 2024). However, catecholamines are inherently less stable than synthetic SABAs and they are COMT-sensitive meaning they are at higher risk of compound degradation over time.

In addition, isoprenaline and adrenaline have been shown to effect antagonist affinity measurements in CRE-SPAP assays in comparison to when salbutamol or terbutaline are the agonists. Thus in CHO-β_2_-WT cells, the affinity of ICI118551 and propranolol are 10-fold lower as measured from a rightward parallel shift of isoprenaline and adrenaline concentration response curves, compared to when salbutamol and terbutaline are the agonists (Baker et al., 2003). The reasons for this remain unclear (and may or may not be related to higher efficacy agonist causing receptor phosphorylation desensitization and internalisation) but is not present for fenoterol, hence fenoterol (inherently more stable and COMT-insensitive) was included in the main study. There have been mixed reports that different β_2_-polymorphisms could affect β_2_-adrenoceptor internalisation (reviewed in Ahles and Engelhardt, 2014), so isoprenaline responses at β_2_-WT and the β_2_-polymorphisms were also investigated.

*^3^H-CGP12177 whole cell binding*

Isoprenaline was confirmed to be a short-acting ligand at all receptors (Supplementary Table S3)

Supplementary Table S3

pK_D_ values, from ^3^H-CGP12177 whole cell binding for isoprenaline and the rightward log shift following washout of isoprenaline. Values are mean ± sem of n separate experiments.

|  | pK_D_ isoprenaline | n | Log shift | n |
| --- | --- | --- | --- | --- |
| CHO-β_2_-WT | 6.58 ± 0.12 | 9 | 2.68 ± 0.16 | 9 |
| CHO-β_2_-gly16 | 6.25 ± 0.06 | 5 | 2.68 ± 0.14 | 5 |
| CHO-β_2_-gln27 | 6.24 ± 0.05 | 5 | 2.79 ± 0.20 | 5 |
| CHO-β_2_-met34 | 6.57 ± 0.06 | 7 | 2.86 ± 0.21 | 6 |
| CHO-β_2_-ile164 | -6.16 ± 0.05 | 5 | 2.51 ± 0.10 | 5 |
| CHO-β_2_-H296K K305D | 6.12 ± 0.03 | 5 | 2.56 ± 0.19 | 5 |
| CHO-β_1_ | 5.96 ± 0.04 | 8 | 2.99 ± 0.10 | 8 |

*CRE-SPAP production*

Given previous findings that antagonist affinity measurements at β_2_-adrenoceptors were different in the presence of catecholamines vs non-catecholamine SABAs, the responses to isoprenaline and the ability of ICI118551 and propranolol to inhibit these responses was examined (Figure S1, Table S2).

Three different concentrations of antagonist were used enabling a Schild plot to be constructed using the following equation:

Log (DR-1) = log [B] – log (K_D_)

where DR (dose ratio) is the ratio of the agonist concentration required to stimulate an identical response in the presence and absence of a fixed concentration of antagonist [B]. These points were fitted to a straight line where a slope of 1 indicates competitive antagonism (Arunlakshana and Schild, 1959).

Isoprenaline responses were inhibited by ICI118551 and propranolol however the pK_D_ values obtained suggested about 10-fold lower affinity for the antagonists compared with the affinity measurements obtained when salbutamol was the agonist. Furthermore, when the Schild slopes are examined, whereas ICI118551 and propranolol appear as competitive antagonists when salbutamol is the agonist (Schild slopes close to 1), the Schild slopes obtained when isoprenaline was the agonist are considerably less than 1 (just as in Baker et al., 2003).

The reason for the difference in antagonist affinity measurement in the presence of isoprenaline compared to salbutamol (and all other agonists Table 3a) remains unknown, however it occurs all β_2_-WT and all β_2_-polymorphic variants.

A reduction in the affinity of ICI118551 was seen for the β_2_-ile164 variant. When isoprenaline was the agonist, this reduction in the affinity of ICI118551, but not propranolol, is also seen in the β_2_-ile164 polymorphism compared to β_2_-WT or the other polymorphisms (as in Tables S1 and S2). Thus for the β_2_-ile164 polymorphic receptor, the affinity of ICI118551 when isoprenaline is agonist is reduced for 2 reasons: because isoprenaline was the agonist (reasons unknown) and because the affinity of ICI118551 is less with this single amino acid change.

Supplementary Table S4

pEC_50_ values and fold over basal for CRE-SPAP production for isoprenaline and pK_D_ values for ICI118551 and propranolol measured from a rightward parallel shift of the isoprenaline concentration response. Schild slopes are also given. Values are mean ± sem of n separate experiments. The salbutamol data is taken from the Tables 3 and Table S2), now with the Schild slopes for ICI118551 and propranolol included.

| Isoprenaline as agonist | | | | | | | | | | | |
| --- | --- | --- | --- | --- | --- | --- | --- | --- | --- | --- | --- |
|  | pEC_50_ isoprenaline | Fold over basal | n | pK_D_ ICI118551 | n | slope | n | pK_D_ propranolol | n | slope | n |
| CHO-β_2_-WT | 7.67 ± 0.08 | 2.71 ± 0.07 | 13 | 8.97 ± 0.08 | 21 | 0.74 ± 0.04 | 7 | 8.87 ± 0.07 | 18 | 0.73 ± 0.03 | 6 |
| CHO-β_2_-gly16 | 7.91 ± 0.18 | 3.66 ± 0.15 | 7 | 8.85 ± 0.15 | 12 | 0.72 ± 0.09 | 4 | 8.48 ± 0.14 | 12 | 0.72 ± 0.05 | 4 |
| CHO-β_2_-gln27 | 7.99 ± 0.19 | 3.13 ± 0.14 | 7 | 8.73 ± 0.16 | 12 | 0.73 ± 0.08 | 4 | 8.41 ± 0.16 | 12 | 0.66 ± 0.08 | 4 |
| CHO-β_2_-met34 | 7.74 ± 0.18 | 4.70 ± 0.18 | 8 | 8.82 ± 0.15 | 12 | 0.76 ± 0.03 | 4 | 8.66 ± 0.11 | 9 | 0.74 ± 0.01 | 3 |
| CHO-β_2_-ile164 | 7.52 ± 0.13 | 3.17 ± 0.16 | 7 | 8.13 ± 0.14** | 12 | 0.67 ± 0.09 | 4 | 8.60 ± 0.18 | 11 | 0.61 ± 0.02 | 3 |
|  |  |  |  |  |  |  |  |  |  |  |  |
| Salbutamol as agonist | | | | | | | | | | | |
|  | pEC_50_ salbutamol | % isoprenaline | n | pK_D_ ICI118551 | n | slope | n | pK_D_ propranolol | n | slope | n |
| CHO-β_2_-WT | 7.53 ± 0.07 | 87.1 ± 1.6 | 23 | 9.77 ± 0.03 | 40 | 0.99 ± 0.02 | 7 | 9.57 ± 0.05 | 31 | 0.99 ± 0.02 | 7 |
| CHO-β_2_-gly16 | 7.93 ± 0.06 | 90.6 ± 3.2 | 16 | 9.87 ± 0.05 | 21 | 1.08 ± 0.05 | 4 | 9.78 ± 0.08 | 21 | 1.02 ± 0.02 | 4 |
| CHO-β_2_-gln27 | 8.04 ± 0.06 | 90.9 ± 2.6 | 15 | 9.83 ± 0.05 | 21 | 0.99 ± 0.04 | 4 | 9.76 ± 0.05 | 20 | 0.99 ± 0.03 | 4 |
| CHO-β_2_-met34 | 7.83 ± 0.10 | 95.9 ± 2.3 | 16 | 9.75 ± 0.06 | 24 | 1.07 ± 0.06 | 4 | 9.74 ± 0.07 | 25 | 1.06 ± 0.02 | 4 |
| CHO-β_2_-ile164 | 7.22 ± 0.08 | 79.3 ± 3.6 | 17 | 8.81 ± 0.06** | 23 | 1.00 ± 0.10 | 4 | 9.46 ± 0.05 | 22 | 0.93 ± 0.02 | 3 |

**p<0.0001 One-way ANOVA with post hoc Newman-Keuls comparing pK_D_ values for ICI118551 with those obtained in the presence of isoprenaline at β_2_-WT and all polymorphic variants. Thus the pK_D_ for ICI118551 in the presence of isoprenaline and salbutamol in β_2_-ile164 is different from that obtained from the β_2_-WT with p<0.0001. The pK_D_ values for propranolol were not different.

Supplementary Figure S1


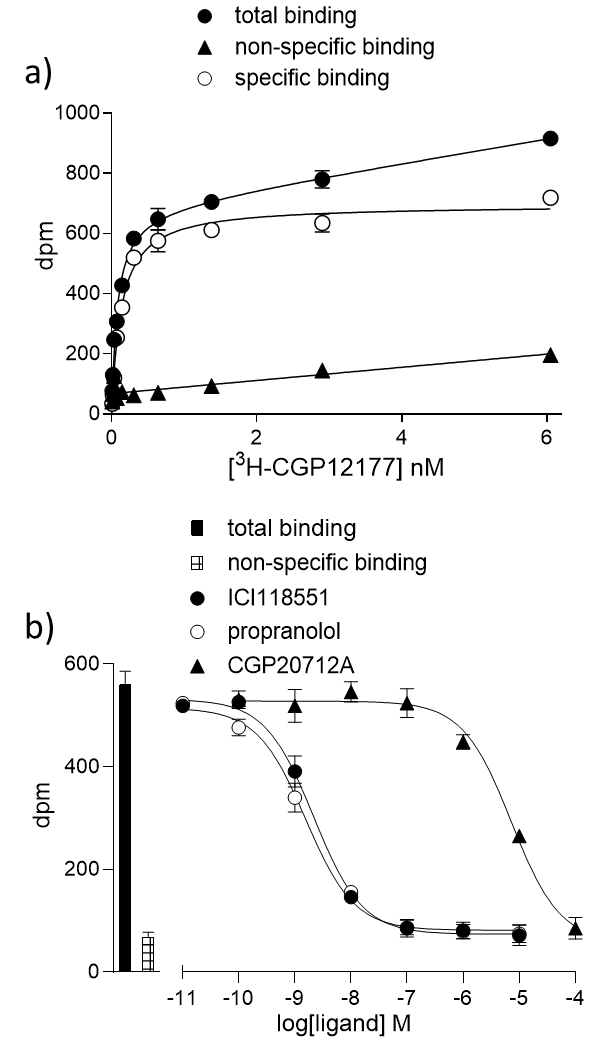


^3^H-CGP12177 whole cell binding in CHO-β_2_ cells a) saturation binding to determined K_D_ value of ^3^H-CGP12177 and b) competition binding used to determined the K_D_ of others ligands showing inhibition of ^3^H-CGP12177 binding by ICI118551, propranolol and CGP20712A. Non-specific binding was determined by 10µM propranolol. The concentration of ^3^H-CGP12177 in b) was 0.49nM. Data points are mean ± s.e.mean of a) quadruplicate determinations and b) triplicate determinations and these single experiments are representative of a) 15 and b) 8 separate experiments.

Supplementary Figure S2


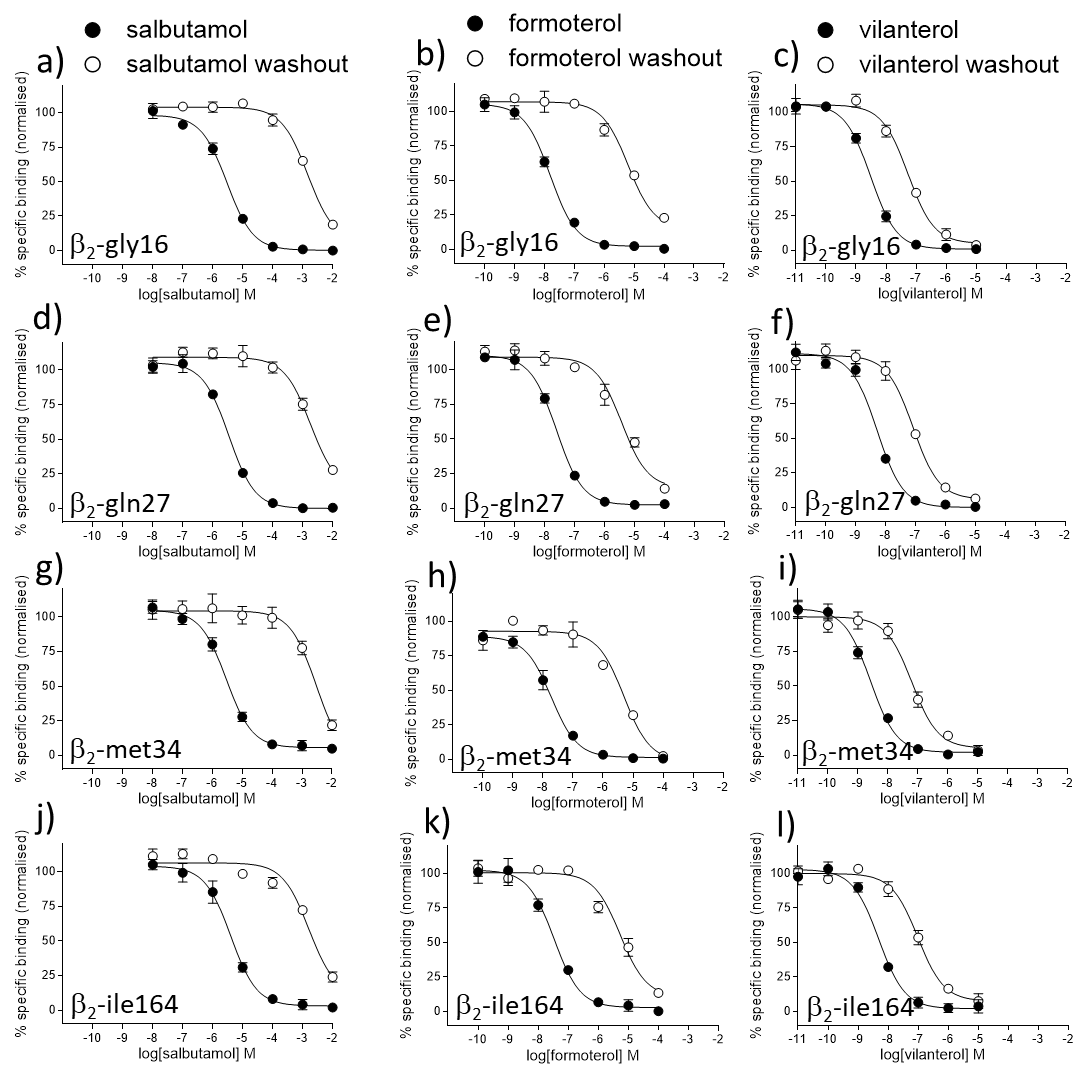


Inhibition of ^3^H-CGP12177 specific binding in whole cells stably expressing each of the naturally occurring β_2_-adrenoceptor polymorphic receptors, a-c) CHO-β_2_-gly16, d-f) CHO-β_2_-gln27, g-i) CHO-β_2_-met34 and j-l) CHO-β_2_-ile164, in response to salbutamol (a, d, g, j), formoterol (b, e, h, k) and vilanterol (c, f, i, l) and following washout of the β_2_-agonists. Non-specific binding was determined by 10µM propranolol and data points are mean ± s.e.mean of triplicate determinations. The concentrations of ^3^H-CGP12177 present in these single experiments was a) 0.57nM, b) 0.67nM, c) 0.48, d) 0.67nM, e) 0.38nM, f) 0.67 nM, g) 0.67nM, h) 0.65nM i) 0.57, j) 0.67nM, k) 0.67nM and l) 0.48nM and they are representative of a) 8, b) 9, c) 10, d) 9, e) 8, f) 10, g) 6, h) 9, i) 11, j) 8, k) 8 and l) 9 separate experiments.

Supplementary Figure S3


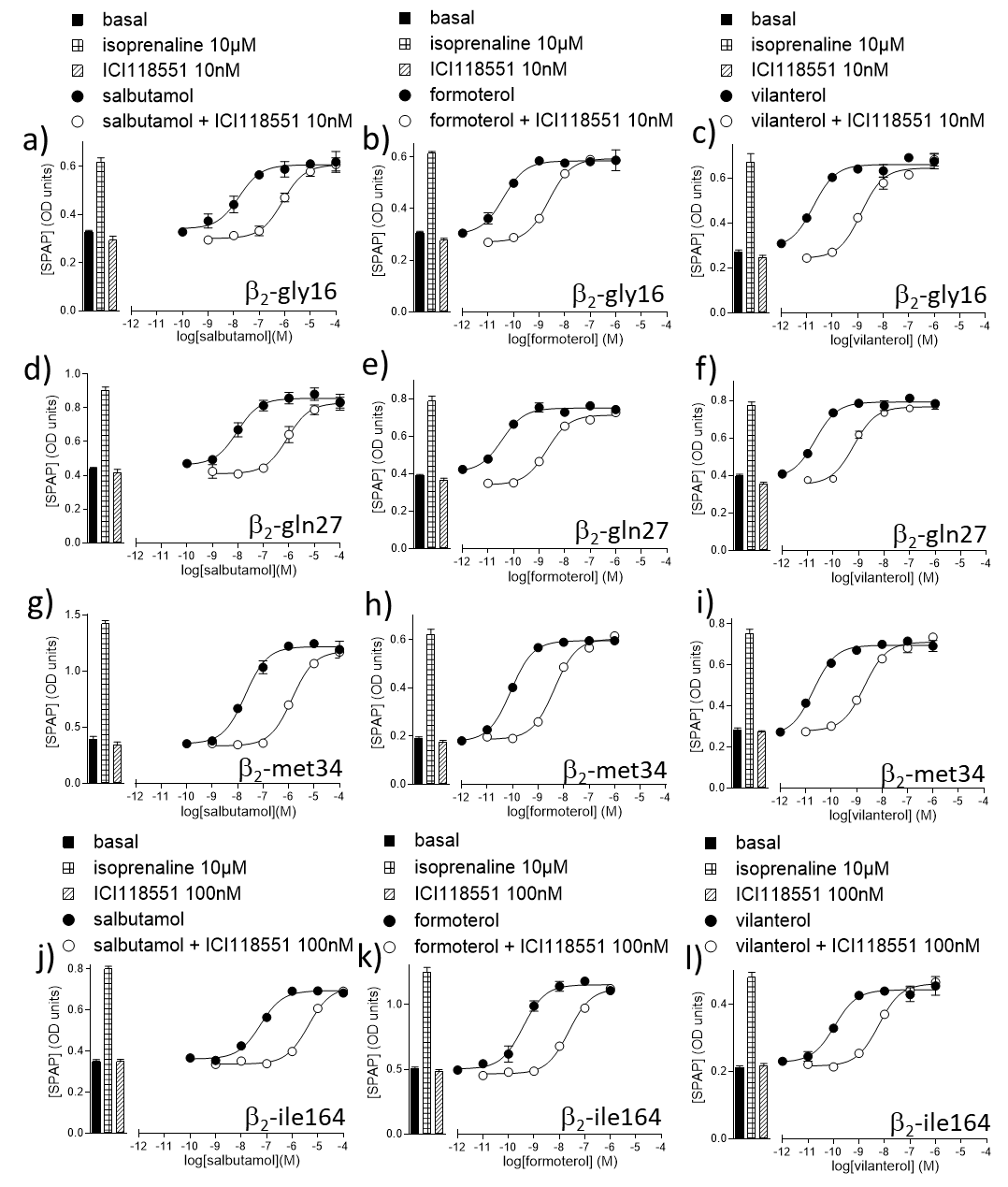


CRE-SPAP production whole cells stably expressing each of the naturally occurring β_2_-adrenoceptor polymorphic receptors, a-c) CHO-β_2_-gly16, d-f) CHO-β_2_-gln27, g-i) CHO-β_2_-met34 and j-l) CHO-β_2_-ile164, in response to salbutamol (a, d, g, j), formoterol (b, e, h, k) and vilanterol (c, f, i, l) in the absence and presence of 10nM ICI118551 (a-i) and 100nM ICI118551 (j-l). Bars represent basal and CRE-SPAP production in response to 10µM isoprenaline or 10nM or 100nM ICI118551 alone. Data points are mean ± s.e.mean of triplicate determinations. These single experiments are representative of a) 16, b) 12, c) 9, d) 15, e) 11, f) 9, g) 16, h) 10, i) 10, j) 17, k) 14 and l) 11 separate experiments.

Supplementary Figure S4


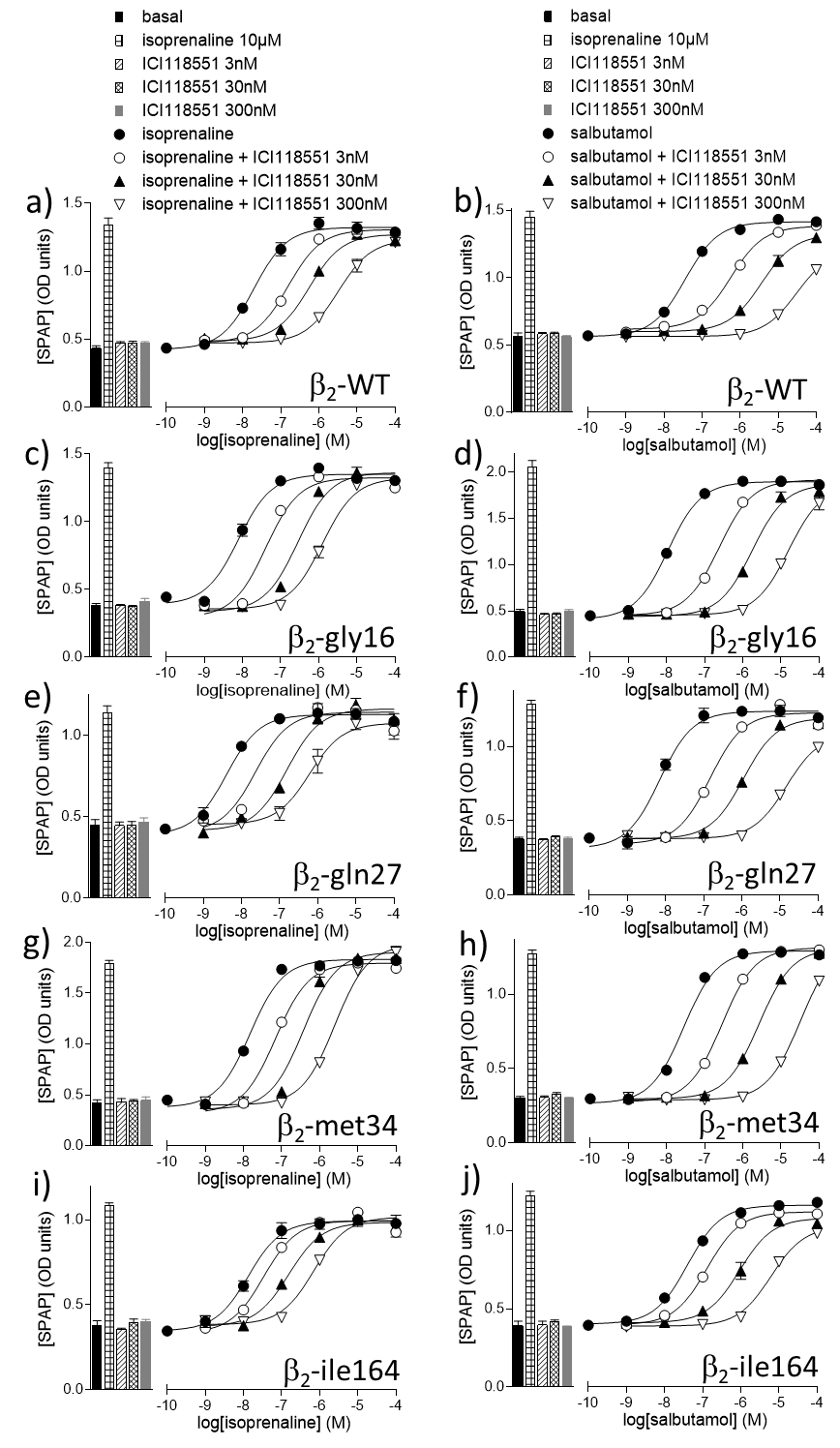


CRE-SPAP production in a-b) CHO-β_2_ cells, c-d) CHO-β_2_-gly16 cells, e-f) CHO-β_2_-gln27 cells, g-h) CHO-β_2_-met34 cells and i-j) CHO-β_2_-ile164 cells in response to isoprenaline (a, c, e, g, i) and salbutamol (b, d, f, h, j) in the absence and presence of 3nM, 30nM or 300 nM ICI118551. Bars represent basal and CRE-SPAP production in response to 10µM isoprenaline or to 3nM, 30nM or 300nM ICI118551 alone. Data points are mean ± s.e.mean of triplicate determinations and these single experiments are representative of a-b) 7 and c-j) 4 separate experiments in each case.

References

Ahles A, Engelhardt S. (2014) Polymorphic variants of adrenoceptors: pharmacology, physiology, and role in disease. Pharmacol Rev. 66(3):598-637. doi: 10.1124/pr.113.008219.

Arunlakshana O, and Schild HO (1959) Some quantitative uses of drug antagonists. Br. J. Pharmacol Chemother. 14: 48-58. doi: 10.1111/j.1476-5381.1959.tb00928.x.

Baker JG. (2005) Sites of action of β-ligands at the human β1-adrenoceptor. J. Pharmacol Exp Ther 313: 1163-1171. doi: 10.1124/jpet.104.082875.

Baker JG (2010) The selectivity of β-adrenoceptor agonists at the human β1, β2 and β3 adrenoceptors. Br. J. Pharmacol 160: 148-161  doi: 10.1111/j.1476-5381.2010.00754.x.

Baker JG, Hall IP, Hill SJ. (2003) Influence of agonist efficacy and receptor phosphorylation on antagonist affinity measurements: Differences between second messenger and reporter gene responses. Mol. Pharmacol. 64: 679-688. doi: 10.1124/mol.64.3.679.

Baker JG, Hall IP, Hill SJ (2004) Temporal characteristics of CRE-mediated gene transcription: requirement for sustained cAMP production. Mol. Pharmacol. 65: 986-998. doi: 10.1124/mol.65.4.986.

Baker JG, Proudman RGW, Hill SJ (2014) Identification of key residues in transmembrane 4 responsible for the secondary, low affinity conformation of the human β1-adrenoceptor. Mol Pharmacol. 85: 811-829. doi: 10.1124/mol.114.091587.

Baker JG, Shaw DE. (2024) Asthma and COPD: A Focus on β-Agonists - Past, Present and Future. Handb Exp Pharmacol. 285:369-451. doi: 10.1007/164_2023_679.

Crompton G. (2006) A brief history of inhaled asthma therapy over the last fifty years. Prim Care Respir J. 2006 Dec;15(6):326-31. doi: 10.1016/j.pcrj.2006.09.002.

Pearce N (2007) Adverse reactions: the fenoterol story. Auckland University Press ISBN: 9781869403744
